# The SARS-CoV-2 protein NSP2 impairs the silencing capacity of the human 4EHP-GIGYF2 complex

**DOI:** 10.1016/j.isci.2022.104646

**Published:** 2022-06-20

**Authors:** Limei Zou, Clara Moch, Marc Graille, Clément Chapat

**Affiliations:** 1 Laboratoire de Biologie Structurale de La Cellule (BIOC), CNRS, Ecole Polytechnique, IP Paris, 91128 Palaiseau, France

**Keywords:** Virology, Molecular biology

## Abstract

There is an urgent need for a molecular understanding of how SARS-CoV-2 influences the machineries of the host cell. Herein, we focused our attention on the capacity of the SARS-CoV-2 protein NSP2 to bind the human 4EHP-GIGYF2 complex, a key factor involved in microRNA-mediated silencing of gene expression. Using *in vitro* interaction assays, our data demonstrate that NSP2 physically associates with both 4EHP and a central segment in GIGYF2 in the cytoplasm. We also provide functional evidence showing that NSP2 impairs the function of GIGYF2 in mediating translation repression using reporter-based assays. Collectively, these data reveal the potential impact of NSP2 on the post-transcriptional silencing of gene expression in human cells, pointing out 4EHP-GIGYF2 targeting as a possible strategy of SARS-CoV-2 to take over the silencing machinery and to suppress host defenses.

## Highlights

• SARS-CoV-2 NSP2 binds the human 4EHP-GIGYF2 complex
• The NSP2/GIGYF2-4EHP interaction involves multiple binding sites
• NSP2 reduces the silencing capacity of GIGYF2
• NSP2 impairs microRNA-mediated silencing

## Graphical abstract

**Figure:**
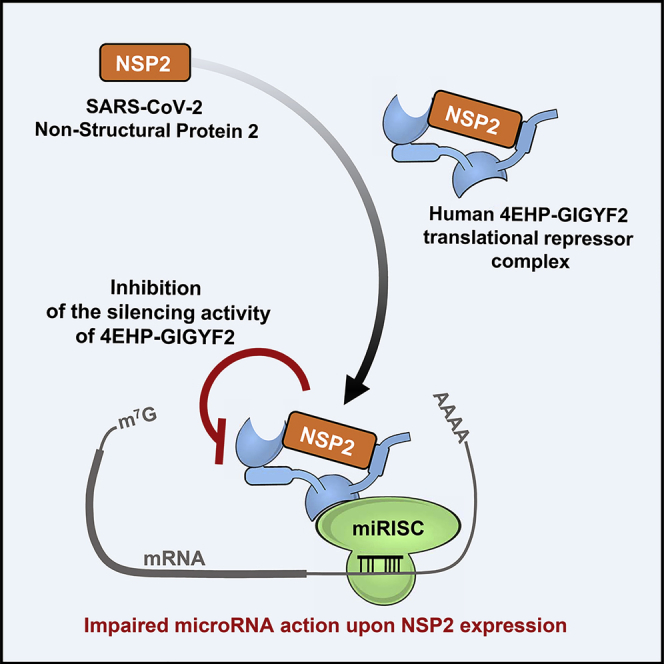

## Introduction

Beta-coronaviruses (β-CoVs) are enveloped RNA viruses that infect a variety of vertebrate hosts, including humans (Cui et al., 2019). In the last decades, two β-CoVs have caused epidemic diseases of the respiratory tract: severe acute respiratory syndrome (SARS-CoV-1) in 2002 (Ksiazek et al., 2003; Drosten et al., 2003) and Middle East respiratory syndrome (MERS) in 2012 (Zaki et al., 2012). A new β-CoV (SARS-CoV-2) emerged in 2019 that is the causative agent of coronavirus disease 2019 (COVID-19) pandemic (Zhou et al., 2020). These three viruses possess single-stranded, positive-sense RNA genomes of nearly 30 kb in length (Lu et al., 2020). The SARS-CoV-2 genome encodes 29 proteins with multiple functions in virus replication and packaging, including 4 structural proteins (the nucleocapsid N, envelope E, membrane M, and spike S proteins), 7 accessory proteins (ORF3a–ORF8) whose functions in SARS-CoV-2 pathogenesis remain largely unknown, and 16 non-structural proteins (NSP1–NSP16) that encode the RNA-directed RNA polymerase, helicase, protease, and other components required for virus replication (for review, see (V'kovski et al., 2021)).

Owing to the urgent need to better understand SARS-CoV-2 biology, several CRISPR-Cas9 and proteomic-based screening campaigns have investigated the landscape of host factors which are targeted by the virus proteome (Hoffmann et al., 2021; Sadegh et al., 2020; Gordon et al., 2020a, 2020b; Davies et al., 2020). These screens identified more than 300 high-confidence protein-protein interactions between human and SARS-CoV-2 proteins, highlighting the intimate connection of SARS-CoV-2 proteins with multiple biological processes, including protein trafficking, transcription, and mRNA translation. Among these interactions, the non-structural protein 2 (NSP2) has been found to interact with key host proteins involved in vesicle trafficking (FKBP15 and WASHC) and mRNA translation (4EHP and GIGYF2) which could be of therapeutic importance (Gordon et al., 2020b; Davies et al., 2020).

NSP2 exists in all coronaviruses studied to date, including SARS-CoV-1, SARS-CoV-2, MERS, and in their closely related β-CoVs infecting mammals. Although its role in the SARS-CoV-2 pathogenicity has not been fully elucidated, the deletion of NSP2 in SARS-CoV-1 attenuates viral growth and RNA synthesis (Graham et al., 2005). Recent findings showed that SARS-CoV-2 NSP2 is undergoing positive nature selection and could be thus essential to the virus (Angeletti et al., 2020; Flores-Alanis et al., 2021). At the structural level, NSP2 has a complex multi-domain topology including an N-terminal domain with a highly conserved zinc binding site, and a C-terminal region rich in β-strands. With the exception of the zinc binding site, NSP2 displays a rapidly evolving surface with the presence of natural variations that could impact host-virus interactions (Ma et al., 2021; Mompean et al., 2021; Gupta et al., 2021; Slavin et al., 2021). Among its host interactors, SARS-CoV-2 NSP2 interacts with the 4EHP-GIGYF2 complex, a key machinery in translational silencing and mRNA decay (Davies et al., 2020; Cornillez-Ty et al., 2009; Gordon et al., 2020a, 2020b). It is worth noting that the NSP2/4EHP-GIGYF2 association is conserved across SARS-CoV-1 and MERS, corroborating a functional importance for β-CoV infection in general (Gordon et al., 2020a; Cornillez-Ty et al., 2009).

The cap-binding eIF4E-Homologous Protein (4EHP) is an integral component of post-transcriptional silencing mechanisms through competing with the eIF4F complex for binding to the mRNA 5′cap (Christie and Igreja, 2021). In complex with the GRB10-interacting GYF (glycine-tyrosine-phenylalanine domain) protein 2 (GIGYF2), the cap-binding activity of 4EHP is required for the optimal translational repression by microRNAs (miRNAs), as well as the RNA-binding proteins ZNF598 and tristetraprolin (TTP) (Morita et al., 2012; Fu et al., 2016; Villaescusa et al., 2009; Cho et al., 2005, 2006; Chapat et al., 2017b; Chen and Gao, 2017). In the case of miRNA-driven silencing, the recruitment of 4EHP-GIGYF2 is initiated by the miRNA-induced silencing complex (miRISC), an assembly of argonaute and TNRC6/GW182 proteins. 4EHP can be physically mobilized through the interaction of the GYF domain of GIGYF2 with a proline-proline-glycine-leucine (PPGL) motif in TNRC6/GW182 (Kryszke et al., 2016; Schopp et al., 2017). At the functional level, this 4EHP/miRNA axis is required to control ERK signaling, as well as to suppress IFN-β production by affecting the *miR-34a*-induced translational silencing of *Ifnb1* mRNA (Zhang et al., 2021; Jafarnejad et al., 2018).

Beyond miRNA action, 4EHP-GIGYF2 also forms a translation inhibitory complex with the RNA-binding protein ZNF598, which functions in ribosome stalling on internally polyadenylated mRNAs during ribosome quality control (Morita et al., 2012; Garzia et al., 2017). ZNF598 is also necessary for the repression of TTP-targeted mRNAs that encode inflammatory cytokines. The 4EHP-GIGYF2-ZNF598 complex binds TTP during an innate immune response in mouse macrophages to control the production of TTP-targeted mRNAs such as TNF-α, Ier3, Csf2, and Cxcl10. In all cases, TNRC6/GW182, ZNF598, and TTP display a comparable binding mode to GIGYF2, namely via the recognition of a proline stretch by the GYF domain of GIGYF2 (Tollenaere et al., 2019; Fu et al., 2016).

A recent genetic screen has revealed that both 4EHP and GIGYF2 are necessary for infection by SARS-CoV-2 *in vitro*, while dispensable for seasonal coronaviruses (Hoffmann et al., 2021). The contribution of 4EHP-GIGYF2 into the pathogenicity of SARS-CoV-2 could therefore originate from their interaction with NSP2. In the present report, we focused on the physical and functional interplays existing between the 4EHP-GIGYF2 complex and the SARS-CoV-2 protein NSP2 in human cells. Combining interaction assays and reporter-based approaches, our data shed light on the negative impact of NSP2 on the 4EHP-GIGYF2-mediated translational silencing of gene expression.

## Results

### NSP2 binds the 4EHP-GIGYF2 complex *in cellulo*

**Figure 1:**
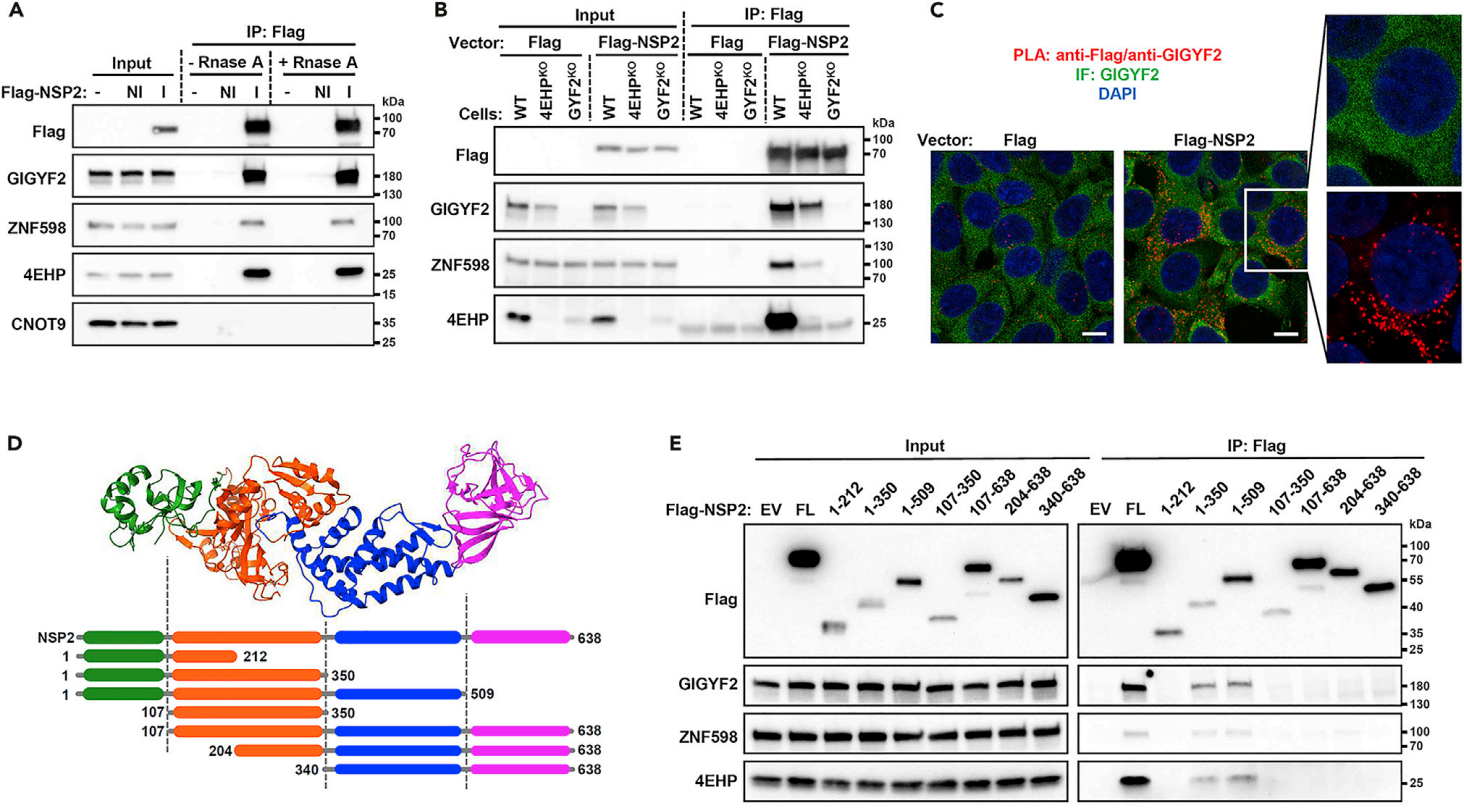
NSP2 interacts with the 4EHP-GIGYF2 complex in the cytoplasm (A) Co-immunoprecipitation (co-IP) between Flag-NSP2 and endogenous 4EHP-GIGYF2. A tetracycline-inducible Flag-NSP2 construct was stably transfected in HEK293 Flp-In T-REx. Extracts from untransfected (−), non-induced (NI), or tetracycline-induced (I) cells were immunoprecipitated with anti-Flag antibody. Total lysates (input) and IP samples were analyzed by Western blot with the indicated antibodies. (B) Flag-NSP2 IP in 4EHP^KO^ or GIGYF2^KO^ cells. Vectors encoding Flag-NSP2, or Flag as control, were transiently transfected in the wild-type (WT), 4EHP^KO^, or GIGYF2^KO^ HEK293 cells, and Flag IPs were performed in RNase A-treated extracts, followed by Western blot with the indicated antibodies. (C) Proximity ligation assay between Flag-NSP2 and endogenous GIGYF2. HEK293T cells were transfected with vector expressing Flag-NSP2, or Flag as control. PLA was performed using anti-Flag and anti-GIGYF2 antibodies. Representative images of PLA (red), along with GIGYF2 immunofluorescence (IF; green) and DAPI (blue) are shown. Z-projection of 3 stacks (0.35 μm each). Scale bars, 5 μm. (D) Representation of the NSP2 protein structure (PDB: 7MSW (Gupta et al., 2021)) and schematic cartoon of Flag-tagged NSP2 truncations used in panel (E). (E) The N-terminal half of NSP2 binds 4EHP-GIGYF2. The indicated constructs were expressed in HEK293T cells and Flag IPs were performed with RNase A-treated extracts. The starting material (Input) and bound fractions were analyzed by Western blot. EV: empty vector; FL: Full length.

Early large-scale studies reported the capacity of SARS-CoV-2 NSP2 to bind the 4EHP-GIGYF2 complex using affinity purification-mass spectrometry (Gordon et al., 2020a, 2020b). To validate the physical association of NSP2 with the GIGYF2-4EHP complex, we first sought to detect their interaction using co-immunoprecipitation (co-IP). An inducible Flag-tagged version of NSP2 was stably expressed in HEK293 Flp-In T-REX cells and co-IPs were performed following tetracycline-induced expression of Flag-NSP2. Lysates prepared from control, non-induced, and induced cells were immunoprecipitated with anti-Flag antibody. Subsequent Western blot (WB) analysis of the co-IP fraction showed that Flag-NSP2 efficiently binds both endogenous GIGYF2 and 4EHP (Figure 1A). This interaction was similarly detected in RNase A-treated lysates, indicating that the NSP2/4EHP-GIGYF2 interaction occurs in an RNA-independent manner. The GIGYF2-associated protein ZNF598 was also found along with NSP2, while CNOT9, a subunit of the CCR4-NOT complex known to bind GIGYF2, was not detected (Ajiro et al., 2009) (Figure 1A). Together, these data demonstrate that NSP2 physically associates with the 4EHP-GIGYF2 complex, as well as their interacting protein ZNF598.

To elucidate which protein is involved in the formation of this complex, GIGYF2- and 4EHP-knockout (KO) HEK293 cells were used to conduct co-IPs with Flag-NSP2. Vectors expressing Flag-NSP2, or Flag as a control, were then transiently transfected in these KO populations, as well as in their wild-type (WT) counterpart. Following Flag IP in the 4EHP^KO^ cells, we observed that the NSP2/GIGYF2 interaction was still detectable, while ZNF598 co-IP was reduced, indicating a plausible contribution of 4EHP in NSP2 binding. By contrast, in the absence of GIGYF2, we could not detect any interaction of NSP2 with either 4EHP or ZNF598 (Figure 1B), supporting a central role of GIGYF2 in NSP2 binding. However, a contribution of 4EHP cannot be excluded since its level is decreased in the GIGYF2^KO^ cells due to a co-stabilization effect (Figure 1B) (Morita et al., 2012).

We then performed immunofluorescence staining and confocal imaging to analyze the subcellular distribution of NSP2. Following transient expression of Flag-tagged NSP2 in WT and GIGYF2^KO^ cells, immunofluorescence staining was performed with antibodies raised against the Flag sequence and endogenous GIGYF2. In WT cells, a diffuse cytoplasmic signal was detected for both Flag-tagged NSP2 and GIGYF2 (Figure S1A), indicating their mutual localization in the cytoplasm. By contrast, the GIGYF2 signal was lost in GIGYF2^KO^ cells while the distribution of Flag-NSP2 remained unchanged (Figure S1A), confirming the specificity of the anti-GIGYF2 antibody. The subcellular localization of NSP2/GIGYF2 interaction was then examined using an *in situ* proximity ligation assay (PLA). PLA was conducted in Flag-NSP2-expressing cells using the anti-Flag and anti-GIGYF2 antibodies. Following confocal imaging, a spot-like signal was abundantly detected in the cytoplasm of HEK293T cells expressing Flag-NSP2, indicating the spatial proximity between endogenous GIGYF2 and Flag-NSP2 (Figure 1C). This PLA signal was not observed in cells transfected with a control plasmid. Similarly, the PLA signal showed a significant reduction when the Flag/GIGYF2 staining was performed in GIGYF2^KO^ cells expressing Flag-NSP2 (Figures S1B and S1C), supporting the specificity of the interaction.

In addition to its diffuse cytoplasmic localization, GIGYF2 is known to be found in P-bodies, a subclass of RNA granules enriched in translationally repressed mRNAs and silencing factors (Amaya Ramirez et al., 2018). We therefore tested whether NSP2 could also be detected in P-bodies by examining the localization of Flag-tagged NSP2 alongside DDX6, a known resident of P-bodies. Using immunofluorescence microscopy, we found that Flag-NSP2 did not form foci that co-localized with DDX6 in HEK293T cells (Figure S1D). Intensity line scans were performed along the DDX6 foci to confirm the absence of relationship between the spatial distribution of NSP2 and P-bodies, thus indicating that NSP2 could preferentially bind the diffuse form of GIGYF2 instead of its P-body-associated counterpart. To confirm this point, we took advantage of the properties of biotinylated isoxazole (b-isox), a compound which selectively precipitates proteins located in RNA granules such as P-bodies and stress granules (Han et al., 2012; Kato et al., 2012) (Figure S1E). Extracts of HEK293T expressing Flag-NSP2 were exposed to 100 μM of b-isox, or DMSO as a mock control. By comparing the level of proteins left in the soluble fraction after precipitation (unbound) with the precipitated fractions (pellet), we confirmed that Flag-NSP2 was not recovered in the pellet whereas a significant proportion of endogenous GIGYF2 was selectively enriched in the b-isox precipitate, alongside DDX6 (Figure S1F). Since NSP2 could not be precipitated by b-isox along with GIGYF2, we concluded that it only binds the diffuse form of GIGYF2 in the cytoplasm.

We next investigated which part of NSP2 binds the 4EHP-GIGYF2 complex. For this purpose, we generated a collection of vectors encoding Flag-tagged truncated versions of NSP2 which were expressed in HEK293T for Flag IP (Figures 1D and 1E). Incremental terminal deletions revealed that the N-terminal half of NSP2 is required for the maximal co-IP of the endogenous 4EHP-GIGYF2 complex as well as ZNF598. While weakly expressed, the NSP2^1−350^ fragment remained the minimal segment which was able to bind 4EHP-GIGYF2. Interestingly, deleting either the N-terminal extremity (fragment NSP2^107−350^) or the middle region (fragment NSP2^1−212^) impaired the NPS2/4EHP-GIGYF2 interaction, indicating a large interaction surface between 4EHP-GIGYF2 and an intact N-terminal half of NSP2 (Figure 1E).

### The NSP2/GIGYF2-4EHP interaction involves multiple binding sites

**Figure 2:**
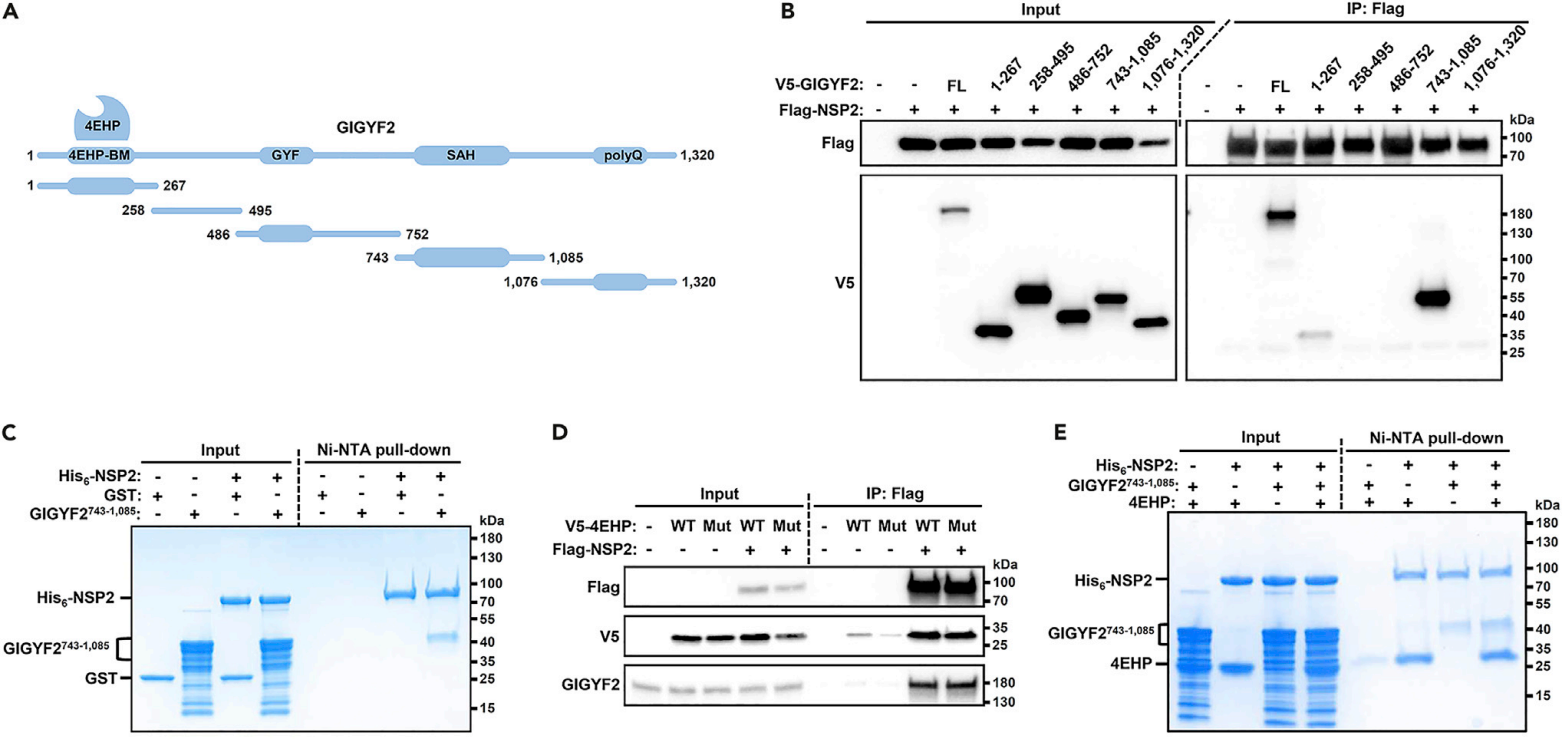
The NSP2/GIGYF2-4EHP interaction involves multiple binding sites (A) Schematic cartoon of the V5-tagged GIGYF2 fragments used in panel (B). 4EHP-BM: 4EHP-binding motif; GYF: glycine-tyrosine-phenylalanine domain; SAH: putative single alpha helix; polyQ: glutamine-rich stretches. (B) Western blot showing the interaction between Flag-NSP2 and two regions in GIGYF2. Vectors expressing Flag-NSP2 and the indicated fragments of V5-tagged GIGYF2 were transiently transfected in HEK293T to perform Flag IP. Extracts were RNase A-treated. Empty vectors were used as negative controls (−). FL: Full-length GIGYF2. (C) Ni-NTA pull-down assay showing the interaction between recombinant His_6_-NSP2 and untagged GIGYF2^743−1,085^. GST served as negative control. The starting material (Input) and bound (Ni-NTA pull-down) fractions were analyzed by SDS-PAGE followed by Coomassie blue staining. (D) Western blot showing the interaction between Flag-NSP2 and V5-4EHP in a GIGYF2-independent manner. RNase A-treated extracts from cells expressing Flag-NSP2 along with V5-4EHP, WT or carrying the W95A substitution (Mut), were used for Flag IP. Inputs and bound fractions were analyzed by Western blotting using the indicated antibodies. Empty vectors served as negative controls (−). (E) Ni-NTA pull-down assay showing the simultaneous interactions between recombinant His_6_-NSP2 and both untagged 4EHP and GIGYF2^743−1,085^. Incubations were performed with the indicated recombinant proteins. The starting material (Input) and bound (Ni-NTA pull-down) fractions were analyzed by SDS-PAGE followed by Coomassie blue staining.

We then sought to delineate which part of the 4EHP-GIGYF2 complex is targeted by NSP2. Human GIGYF2 is a 150 kDa scaffolding protein composed of several domains interspaced by intrinsically disordered regions. These include the 4EHP-binding domain at the N-terminus, the so-called GYF domain, a putative single alpha-helix (SAH), and many glutamine-rich stretches (polyQ) at the C-terminus (Figure 2A) (Peter et al., 2017; Suveges et al., 2009; Morita et al., 2012; Kofler and Freund, 2006). V5-tagged fragments of GIGYF2 were designed to isolate these features, and expressed along with Flag-NSP2 for Flag IP (Figures 2A and 2B). Our WB analysis of the IP fractions revealed that NSP2 binds two distinct segments of GIGYF2, namely the central putative SAH region (residues: 743–1,085), and to a smaller extent, the N-terminal extremity containing the 4EHP-binding motif (residues: 1–267; Figure 2B). The strong interaction between NSP2 and GIGYF2^743−1,085^ prompted us to test their direct association. For this, full-length hexahistidine (His_6_)-tagged NSP2 and glutathione S-transferase (GST)-fused GIGYF2^743−1,085^ were recombinantly expressed in *Escherichia coli* (*E. coli*) and individually purified to perform a His pull-down assay. Because the untagged GIGYF2^743−1,085^ fragment appeared to be unstable following our two-step purification process, we tested whether this recombinant protein could be recognized by an anti-GIGYF2 antibody raised against the 756–1,104 region. Our Western blot assay confirmed that a predominant ∼42-kDa band corresponding to an intact GIGYF2^743−1,085^ is specifically detected by the anti-GIGYF2 antibody (Figure S2A). Following incubation of His_6_-NSP2 with untagged GIGYF2^743−1,085^ on a Ni-NTA resin, analysis of the eluates by SDS-PAGE and Coomassie blue staining revealed a specific retention of GIGYF2^743−1,085^ with His_6_-NSP2, confirming their direct interaction *in vitro* (Figure 2C). Western blot analysis of these samples confirmed that the retained band is specifically recognized by the anti-GIGYF2 antibody (Figure S2B). GST alone was used as control and did not show any pull-down by His_6_-NSP2, supporting the specificity of the NSP2-GIGYF2 interaction. We also successfully detected this interaction when both His_6_-NSP2 and GST-GIGYF2^743−1,085^ were co-expressed in *E. coli*. In this case, a GST pull-down assay was performed using an *E. coli* lysate and a specific His_6_-NSP2 retention on GST-GIGYF2^743−1,085^-bound beads was detected by both Coomassie blue staining and Western blot (Figure S2C). Similarly, we sought to test the capacity of His_6_-NSP2 to bind the N-terminal region of GIGYF2 (residues: 1–267), but failed to obtain this recombinant GIGYF2 fragment with a sufficient yield and solubility (data not shown).

Our co-IP experiments pointed out the 1–350 region of NSP2 as the minimal segment required to bind GIGYF2 *in cellulo* (Figure 1E). In attempt to narrow down this region, we generated three recombinant His_6_-tagged fragments of NSP2 covering the 1–350 region (residues: 1–115, 107–212, and 204–350) to test their direct interaction with GIGYF2^743−1,085^. Using a His pull-down assay, we found that none of these fragments successfully retained GIGYF2^743−1,085^, with the exception of full-length His_6_-NSP2 (Figure S2D), confirming a large interaction surface between NSP2 and GIGYF2. Because an interaction was detected between NSP2 and the 4EHP-binding region of GIGYF2 (residues: 1–267) by co-IP, we also speculated that 4EHP could bind NSP2 independently of GIGYF2. To test this, we used a V5-tagged version of 4EHP carrying the W95A substitution which is known to disrupt its interaction with GIGYF2 (Peter et al., 2017). HEK293T cells were co-transfected by vectors encoding Flag-NSP2 and V5-4EHP^W95A^, or its WT counterpart. Using Flag IPs, we found that both WT and W95A versions of 4EHP were co-immunoprecipitated by NSP2 to a comparable extent (Figure 2D), indicating that 4EHP can bind NSP2 independently of GIGYF2. This result prompted us to evaluate their direct association *in vitro*. As performed with GIGYF2^743−1,085^, full-length GST-fused 4EHP was expressed in *E. coli* and the purified untagged protein was incubated with His_6_-NSP2 to perform a His pull-down assay. In agreement with our co-IP results, we observed a specific binding of 4EHP to His_6_-NSP2, thus confirming their interaction in a GIGYF2-independent manner (Figure S2E). Conversely, we found that NSP2 can simultaneously bind both 4EHP and GIGYF2^743−1,085^ since the latter two were retained in our His_6_-NSP2 pull-down when all three recombinant proteins were incubated at the same time (Figure 2E).

Gupta et al. recently reported that a natural variant of NSP2 carrying the G262V/G265V substitutions showed a reduced interaction with 4EHP-GIGYF2 by affinity purification-mass spectrometry (Gupta et al., 2021). We therefore tested whether these G to V substitutions could impact the integrity of the NSP2/4EHP-GIGYF2 complex by co-IP and *in vitro* interaction assays. Our co-IP experiment with a Flag-tagged NSP2 harboring the G262V/G265V variation confirmed that substituting G262 and G265 with valines reduced its interaction with endogenous GIGYF2, 4EHP, and ZNF598 (Figure S3A). By contrast, when introduced in the His_6_-NSP2 recombinant protein, these two substitutions did not change the capacity of NSP2 to retain both recombinant 4EHP and GIGYF2^743−1,085^ in a His pull-down assay (Figure S3B). Nevertheless, it is worth noting that the direct contribution of the G262 and G265 residues of SARS-CoV-2 NSP2 in binding 4EHP-GIGYF2 remains unclear since both are not found in SARS-CoV-1 and MERS-CoV while the NSP2/4EHP-GIGYF2 interaction is detected across these two β-CoVs (Figure S3C). Overall, these data suggest that the mode of interaction between NSP2 and the intact 4EHP-GIGYF2 complex involves multiple interaction interfaces, and may be more sophisticated *in cellulo* than in our *in vitro* binding assays.

### NSP2 reduces the silencing capacity of GIGYF2

**Figure 3:**
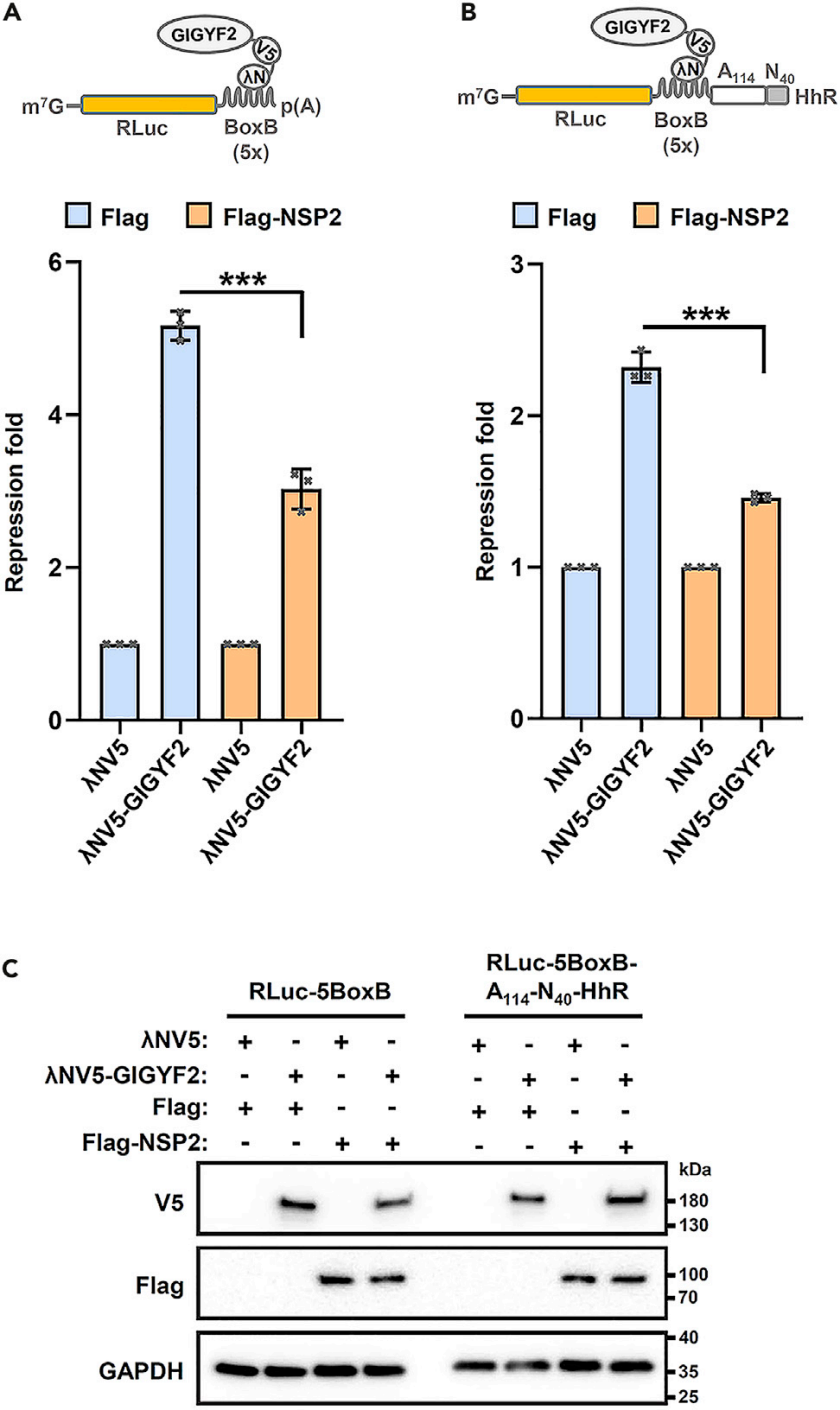
NSP2 decreases the silencing capacities of GIGYF2 *in cellulo* (A) Artificial tethering of GIGYF2 to the 3′ UTR of a reporter mRNA. The upper panel shows a schematic of the λN/BoxB tethering assay with the RLuc-5boxB reporter construct. Recruitment of GIGYF2 to the Renilla luciferase (RLuc) mRNA was mediated by the fused λN peptide. RLuc luminescence was normalized against firefly luciferase (FLuc) level, and repression fold was calculated by dividing the relative luciferase activity of the cells transfected with the control pCI-λNV5 vector (λNV5) by the luciferase activity of λN-GIGYF2-expressing cells. The mean values (±SD) from three independent experiments are shown and the p value was determined by two-tailed Student’s t-test: (∗∗∗) p < 0.001. (B) Artificial tethering of GIGYF2 to the 3′ UTR of a reporter mRNA which is refractory to deadenylation. The upper panel shows a schematic of the RLuc-5boxB-A_114_-N_40_-HhR reporter. HEK293T cells were co-transfected with vectors expressing either λNV5-GIGYF2, or λNV5 as a control, along with RLuc-5boxB-A_114_-N_40_-HhR and FLuc. Vectors encoding Flag-NSP2 or Flag (empty vector) were also added in the transfection mixture. RLuc luminescence was normalized against the FLuc level and analyzed as in (A). (∗∗∗) p < 0.001 (two-tailed Student’s t-test). (C) Extracts from the HEK293T cells used in (A) and (B) were analyzed by Western blot with the indicated antibodies. GAPDH was used as a loading control.

The role of 4EHP-GIGYF2 as a translational repressor has been described in various contexts (for review, see (Christie and Igreja, 2021)). Having shown that NSP2 directly targets the 4EHP-GIGYF2 complex through at least two contact points, namely the region 743–1,085 of GIGYF2 and 4EHP itself, we wished to assess whether the silencing capacity of 4EHP-GIGYF2 could be altered by NSP2. To address this point, we used the λN-BoxB tethering approach in HEK293T cells (Baron-Benhamou et al., 2004; Pillai et al., 2004). A *Renilla* luciferase reporter mRNA containing five BoxB sequences (RLuc-5BoxB) in the 3′ untranslated region (3′UTR) was co-expressed with a plasmid expressing V5-tagged GIGYF2 fused to a λN peptide, which has a high affinity for the BoxB sequences (Figure 3A). The silencing capacity of GIGYF2 was assessed following the measurement of luciferase activity in HEK293T expressing Flag-NSP2 or Flag as control. The expression of Flag-NSP2 and λN-GIGYF2 was also verified by WB (Figure 3C). As expected, we observed that the recruitment of GIGYF2 to the 3′ UTR markedly reduced luciferase activity in control cells (5-fold repression). Interestingly, this λN-GIGYF2-mediated repression was reduced upon expression of Flag-NSP2 (3-fold repression), indicating an inhibitory effect of NSP2 on GIGYF2 function in silencing (Figure 3A).

Several studies have reported that GIGYF2 has two distinct mechanisms of repression: one is 4EHP-dependent and affects translation; the other is 4EHP-independent and involves the deadenylase activity of the CCR4-NOT complex (Amaya Ramirez et al., 2018). Our co-IP data showed that NSP2 did not interact with CCR4-NOT (Figure 1A), suggesting that it should preferentially impair the 4EHP-dependent activity of GIGYF2. To test this, λN-GIGYF2 was tethered to a RLuc-5BoxB mRNA containing a self-cleaving hammerhead ribozyme (HhR) at the 3′-end to generate a poly(A) stretch of 114 nucleotides, followed by 40 nucleotides to block CCR4-NOT-dependent deadenylation (RLuc-5BoxB-A_114_-N_40_-HhR), as previously described (Chapat et al., 2017b). Tethering GIGYF2 to RLuc-5BoxB-A_114_-N_40_-HhR induced a 2.2-fold repression of this reporter in cells transfected with a control vector (Figure 3B). Owing to its inability to encounter mRNA deadenylation, the silencing magnitude of this reporter remained ∼40% lesser than the one induced when GIGYF2 is tethered to RLuc-5BoxB. By contrast, we observed that NSP2 expression decreased the GIGYF2-mediated repression of the reporter to 1.4-fold (Figure 3B), indicating a specific role of NSP2 in blocking the deadenylation-independent silencing capacity of GIGYF2.

### NSP2 impairs the contribution of 4EHP-GIGYF2 into miRNA-mediated silencing

**Figure 4:**
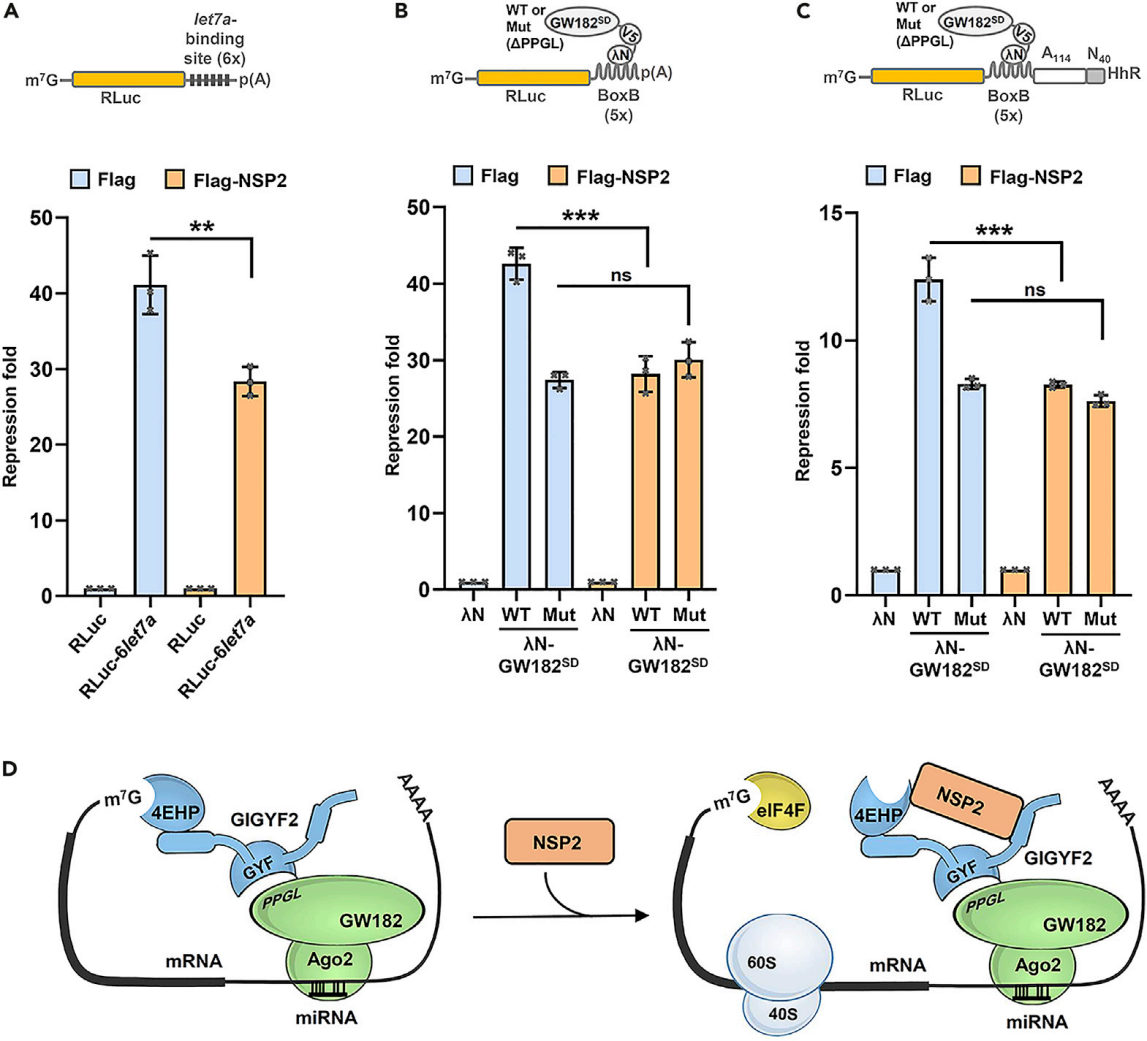
NSP2 impairs miRNA-mediated silencing (A) NSP2 decreases *let7a*-mediated silencing. The upper panel shows a schematic of the RLuc-*6let7a* reporter mRNA. HEK293T cells were co-transfected with RLuc or RLuc-*6let7a* plasmids, along with FLuc construct to account for variations in transfection efficiency. Vectors encoding Flag-NSP2 or Flag (empty vector) were also added in the transfection mixture. Repression fold was calculated by dividing the relative luciferase activity of the cells transfected with the RLuc vector by the luciferase activity of RLuc-*6let7a* expressing cells. Error bars indicate ±SD (n = 3). (∗∗) p < 0.01 (two-tailed Student’s t-test). (B) Artificial tethering of GW182^SD^ to the 3′ UTR of a reporter mRNA. The upper panel shows a schematic of the λN/BoxB tethering assay with the RLuc-5boxB reporter construct. HEK293T cells were co-transfected with vectors expressing either λNV5- GW182^SD^, WT, or a ΔPPGL mutant (Mut), or λNV5 as a control, along with RLuc-5boxB and FLuc. Vectors encoding Flag-NSP2 or Flag (empty vector) were also added in the transfection mixture. RLuc luminescence was normalized against the FLuc level. The mean values (±SD) from three independent experiments are shown and the p value was determined by two-tailed Student’s t-test: (ns) non-significant, (∗∗∗) p < 0.001. (C) Artificial tethering of GW182^SD^ to the 3′ UTR of a reporter mRNA which is refractory to deadenylation. The upper panel shows a schematic of the RLuc-5boxB-A_114_-N_40_-HhR reporter. Transfections were performed as in (B), except that GW182^SD^ was tethered on the RL-5*boxB*-A_114_-N_40_-HhR reporter. Data are presented as mean ± SD (n = 3). (∗∗∗) p < 0.001 (two-tailed Student’s t-test). (D) Model of NSP2-mediated negative regulation of miRNA function. In absence of NSP2 (left panel), miRISC recruits the 4EHP-GIGYF2 complex to effect translational silencing of the targeted mRNA. Following NSP2 expression (right panel), the function of 4EHP-GIGYF2 is physically targeted by NSP2 and its silencing capacity is impaired. The assembly of this complex subsequently alters the magnitude of miRNA-induced silencing.

The activity of the 4EHP-GIGYF2 complex is required for the optimal translation repression driven by several pathways in a large range of cellular processes (Christie and Igreja, 2021). In particular, the miRNA-induced translational repression is affected by the 4EHP-GIGYF2 complex in human cells (Chen and Gao, 2017; Chapat et al., 2017b; Schopp et al., 2017). To evaluate the impact of NSP2 in miRNA-mediated silencing, we transiently transfected HEK293T cells with a *Renilla* luciferase construct either lacking (RLuc), or containing six bulged *let7a* miRNA-binding sites in its 3′ UTR (RLuc-*6let7a*), together with a firefly luciferase construct (FLuc) as a transfection control (Figure 4A). Normalized RLuc activity was markedly reduced by the presence of *let7a*-binding sites, with ∼40-fold repression in cells expressing a control vector. By contrast, expression of NSP2 resulted in a decreased repression magnitude of the RLuc-*6let7a* reporter compared to RLuc (∼30-fold), indicating a reduced *let7a*-mediated silencing in the presence of NSP2 (Figure 4A).

Evidence suggests that the 4EHP-GIGYF2 complex contributes to miRNA action through the binding of GIGYF2 to a proline-rich motif (PPGL) in the silencing domain (SD) of GW182, the scaffolding protein of miRISC (Schopp et al., 2017). To investigate whether NSP2 could impair GW182-mediated silencing, the silencing domain of GW182 was artificially tethered to the RLuc-5BoxB reporter in control and Flag-NSP2-expressing cells. We found that tethering GW182^SD^ induced a ∼40-fold repression of the RLuc-5BoxB level in the control cells, while this silencing effect was reduced in NSP2-expressing cells (∼30-fold; Figure 4B). As a control, a GW182^SD^ mutant carrying a deletion of the GIGYF2-binding motif (ΔPPGL) was used to evaluate the contribution of GIGYF2. In control cells, tethering the ΔPPGL mutant of GW182^SD^ engendered a silencing of RLuc-5BoxB that is ∼35% lesser than the one induced by tethering WT GW182^SD^, confirming the importance of the GIGYF2/PPGL interaction for the GW182-mediated silencing. Tethering this ΔPPGL mutant in cells expressing Flag-NSP2 showed that its silencing activity is not affected by NSP2, indicating that NSP2 action on GW182^SD^-mediated silencing is exerted through targeting GIGYF2 (Figure 4B).

Tethering GW182^SD^ to an mRNA is known to lead to translational repression, deadenylation, and degradation of the target mRNA, while 4EHP-GIGYF2 was only shown to participate to translational repression (Jonas and Izaurralde, 2015; Schopp et al., 2017). To uncouple 4EHP-GIGYF2-driven translational repression from mRNA destabilization effects, GW182^SD^ was tethered to the RLuc-5BoxB-A_114_-N_40_-HhR reporter in control and Flag-NSP2-expressing cells. In this case, the GW182^SD^-mediated repression was reduced to ∼12-fold in control cells due to the inability of RLuc-5BoxB-A_114_-N_40_-HhR to encounter mRNA deadenylation, and to ∼8-fold when the ΔPPGL mutant was used (Figure 4C). Upon NSP2 expression, the silencing activity of GW182^SD^ was decreased to ∼8-fold but remains unchanged with its ΔPPGL version, confirming a specific alteration of the 4EHP-GIGYF2-driven translational repression by NSP2. Altogether, these results indicate that the GIGYF2-dependent repressive activity of GW182^SD^ is partially altered upon expression of NSP2 in human cells.

## Discussion

Upon infection, SARS-CoV-2 impairs splicing, export, translation, and degradation of host mRNAs (Finkel et al., 2021; Banerjee et al., 2020). Here, we present evidence to support a new layer of complexity in the post-transcriptional alteration of the host transcriptome by SARS-CoV-2. We propose that SARS-CoV-2 NSP2 directly targets the 4EHP-GIGYF2 complex to decrease its silencing capacity (Figure 4D). While our model lacks the context of other viral proteins that would be present in a bona fide infection, this mechanism could nonetheless unveil the impact of NSP2 on the post-transcriptional silencing of gene expression of human cells, pointing out 4EHP-GIGYF2 targeting as a possible strategy of SARS-CoV-2 to take over the silencing machinery and to suppress host defenses. Further studies in a more physiological context, such as lung/airway cell lines or SARS-CoV-2-infected samples, should help resolve this conundrum.

How does NSP2 impair 4EHP-GIGYF2 function? Combining co-IP experiments and *in vitro* binding assays with recombinant proteins, we concluded that NSP2 uses its N-terminal region encompassing its conserved zinc finger domain, to interact with the 4EHP-GIGYF2 complex. Our pull-down assays indicate the direct interaction of NSP2 with both 4EHP and two domains from GIGYF2, confirming a sophisticated mode of binding in cellulo. While we searched for the minimal region of NSP2 required for these interactions, we failed to narrow down a fragment smaller than the 1–350 region since truncations at both extremities of this domain abrogate its binding to 4EHP-GIGYF2 (Figures 1E and S2D). Nevertheless, this remains in agreement with Gupta et al. who pointed out that the G262V and G265V mutations located within this region of NSP2 reduced binding to 4EHP-GIGYF2 (Gupta et al., 2021). This natural variation occurs in a poorly conserved patch in NSP2 that is subsequently becoming more hydrophobic due to the G to V substitution. It is worth noting that G262 and G265 are not conserved across the SARS-CoV-1 and MERS-CoV (Figure S3C), while the NSP2/4EHP-GIGYF2 interaction exists among these viruses. The direct contribution of the G262/G265 residues of NSP2 in binding 4EHP-GIGYF2 is therefore questionable. It is tempting to speculate that the G to V variation could rather increase NSP2’s affinity for host interactors that outcompete 4EHP-GIGYF2. This point is supported by the affinity purification-mass spectrometry made by Gupta et al., showing that the G262V/G265V variation increases the affinity of NSP2 for factors such as the mitochondrial protein UQCRC1, or the actin-nucleation-promoting protein WASHC5 (Gupta et al., 2021). This could explain why the G262V/G265V variation reduces the NSP2/4EHP-GIGYF2 interaction in cellulo, but not in our *in vitro* pull-down assay lacking the context of other host interactors of NSP2. Further investigation will be needed to fully resolve the structural basis of the NSP2/4EHP-GIGYF2 complex and thus elucidate NSP2 action on translation silencing. In particular, it now remains to be determined whether NSP2 binding induces conformational changes in 4EHP-GIGYF2 that impair either the cap-binding pocket of 4EHP, or influence the recruitment of GIGYF2’s co-factors such as CCR4-NOT and DDX6.

Global measurement of miRNA action showed that translational repression accounts for 6%–26% of the silencing of each mRNA target in mammalian cells, and 4EHP-GIGYF2-mediated translational repression is observed at early time points of the silencing process (Schopp et al., 2017; Eichhorn et al., 2014). Consistent with these observations, the impact of NSP2 remains mild on the *let7a*-targeted RLuc reporter (Figure 4A). The latter is also known to underestimate the contribution of the translational repression to the silencing process since mRNA destabilization is the dominant effect of miRNA-mediated silencing at steady state (Eichhorn et al., 2014; Bethune et al., 2012). Our tethering assays with GW182^SD^ have proven helpful to overcome this limitation. The derepression of the GW182^SD^-induced silencing of RLuc-5BoxB-A_114_-N_40_-HhR upon deletion of the PPGL motif supports the fact that our tethering assay faithfully recapitulates the contribution of GIGYF2 into miRNA-induced translation repression. Consistently, these tethering assays showed that the silencing capacity of GW182^SD^ upon NSP2 expression equals the one of its ΔPPGL version in control cells (Figures 4B and 4C), indicating that the contribution of 4EHP-GIGYF2 into GW182^SD^-mediated translation repression is fully targeted by NSP2. Indeed, this impact of NSP2 will need to be investigated in further more physiological studies using endogenous miRNAs and transcripts.

At the moment, it is uncertain what the functional interplay between SARS-CoV-2 infection and miRNA-mediated silencing is in human cells. Host miRNAs are known to be produced as a part of antiviral response to counteract the infection by targeting viral transcripts, although SARS-CoV-2 infection was recently shown to have minimal impact on the miRNA repertoire of its host cell (Cullen, 2006; Bruscella et al., 2017; Pawlica et al., 2021). Computational analyses have predicted the presence of many putative miRNA-binding sites on the SARS-CoV-2 genome, suggesting that the SARS-CoV-2 genome could be actively targeted by host miRNAs (Xie et al., 2021; Arisan et al., 2020; Chow and Salmena, 2020). Particularly worth mentioning is the work of Xie et al. which recently identified *let-7*-binding sites in the coding sequence of S and M proteins of SARS-CoV-2 genome, and experimentally confirmed that *let-7* blocks SARS-CoV-2 replication by targeting S and M proteins (Xie et al., 2021). Through the NSP2/4EHP-GIGYF2 axis, SARS-CoV-2 could therefore escape from the host defense system by impairing the function of the effector machinery of miRNAs. The recent discovery that 4EHP and GIGYF2 are needed for infection by SARS-CoV-2 could reinforce this idea, although further research will be required to test this possibility (Hoffmann et al., 2021).

While the silencing capacity of miRISC is partially impeded upon NSP2 expression, there is no guarantee that miRNA action is the prime target of NSP2. The activity of 4EHP-GIGYF2 is mobilized by several pathways, one of which may be more affected than miRNAs. These pathways include TTP- and ZNF598-mediated mRNA silencing, as well as the repression of mRNAs with altered ribosome activity or premature termination codons as part of the nonsense-mediated mRNA decay pathway (Christie and Igreja, 2021). Future studies are thus mandatory to evaluate the potential impact of NSP2 in modulating these processes in human cells. In the case of miRNA, the alteration of *let-7a*-mediated inhibition by NSP2 could be extrapolated to other miRNAs whose action relies on 4EHP, such as *miR-145* or *miR-34a* (Jafarnejad et al., 2018; Zhang et al., 2021). Recent evidence demonstrated that the 4EHP/*miR-34a* axis is required for the translational repression of mRNAs encoding IFN-β through targeting the 3′UTR of *Ifnb1* mRNA (Zhang et al., 2021). Beyond miRNA, 4EHP-GIGYF2 also controls the production of TTP-targeted mRNAs that encode inflammatory cytokines such as TNF-α and IL-8 (Fu et al., 2016; Tollenaere et al., 2019). In this context, a possible consequence of NSP2 expression could be the overproduction of early response pro-inflammatory cytokines. Exploring this point would be of utmost importance since impaired type I interferon activity and inflammatory responses are detected in patients with severe COVID-19 (Hadjadj et al., 2020). To examine whether NSP2 could impact the function of 4EHP in regulating IFN-β expression, we expressed NSP2 along with a reporter construct containing the 3′ UTR of *Ifnb1* mRNA into HEK293T cells (Figure S4A). Remarkably, the reporter expression was repressed ∼2.9-fold in control cells, but only ∼1.6-fold in NSP2-expressing cells, indicating that NSP2 could potentially unbalance the production of IFN-β through the *Ifnb1* 3′ UTR (Figure S4B). With this in mind, further investigations into whether the NSP2/4EHP-GIGYF2 axis can dysregulate sustained cytokine production may therefore prove useful.

In conclusion, our study raises the possibility that SARS-CoV-2 could target the human 4EHP-GIGYF2 complex to selectively modulate its capacity to effect translation repression. Our model may represent a novel framework to investigate the mechanisms underlying the pathogenicity of SARS-CoV-2 based on the interaction of NSP2 with 4EHP-GIGYF2. Ultimately, we hope that this study will be a primer for further more physiological research to evaluate the generalizability of our model.

### Limitations of the study

Although our work depicts how the 4EHP-GIGYF2 complex is compromised by the SARS-CoV-2 protein NSP2, it is not without limitations. The molecular mechanism described in this article comes from *in vitro* studies using the HEK293T cells, which were chosen to dissect the NSP2/4EHP-GIGYF2 interaction on the basis of their capacity to express both NSP2 and 4EHP-GIGYF2 at a high level. However, our model will need to be validated in a more physiological context, such as lung/airway cell lines or SARS-CoV-2-infected samples, to assess the functional relevance of this interaction. In this sense, whether other SARS-CoV-2 proteins interact with NSP2 and thereby prevent its interaction with GIGYF2/4EHP cannot be dismissed. Our conclusion that NSP2 impairs miRNA action is mainly based on the use of artificial reporters (miRNA and tethering assays). To date, these assays have proven invaluable in dissecting the functions of numerous silencing factors in human cells. Nevertheless, our data will need to be complemented by follow-up studies to investigate the impact of NSP2 on endogenous transcripts. Future investigations that employ transcriptomic analysis of the NSP2-mediated post-transcriptional alterations will undoubtedly extend our view on the repertoire of endogenous miRNA/mRNA pairs which are affected by NSP2. Another potential limitation of our study is that we did not explore potential mechanisms for NSP2-mediated inhibition of 4EHP-GIGYF2 and how they could cause the pathogenicity of SARS-CoV-2. Future studies will be needed to dissect the molecular basis of this mechanism using additional models of coronavirus infection.

## STAR★Methods

### Key resources table

**Table:** 

REAGENT or RESOURCE	SOURCE	IDENTIFIER
**Antibodies**		

Rabbit anti-GIGYF2	Proteintech	Cat# 24790-1-AP; RRID:AB_2879727
Rabbit anti-DDX6	Proteintech	Cat# 14632-1-AP; RRID:AB_2091264
Rabbit anti-CNOT9	Proteintech	Cat# 22503-1-AP; RRID:AB_11232413
Rabbit anti-eIF4E2/4EHP	Proteintech	Cat# 12227-1-AP; RRID:AB_10642945
Mouse anti-GAPDH	Proteintech	Cat# 60004-1-Ig; RRID:AB_2107436
Rabbit anti-ZNF598	Thermo Fisher Scientific	Cat# 703,601; RRID:AB_2815335
Mouse anti-Flag M2	Sigma-Aldrich	Cat# F1804; RRID:AB_262044
Mouse anti-V5 tag	Thermo Fisher Scientific	Cat# R960-25; RRID:AB_2556564
Mouse anti-6xHis	Thermo Fisher Scientific	Cat# MA1-21315-HRP; RRID:AB_2536989
Goat anti-Rabbit IgG (H + L) Cross-Adsorbed Secondary Antibody, Alexa Fluor™ 488	Thermo Fisher Scientific	Cat# A-11008; RRID:AB_143165
Goat anti-Mouse IgG (H + L) Cross-Adsorbed Secondary Antibody, Alexa Fluor 594	Thermo Fisher Scientific	Cat# A-11005; RRID:AB_2534073
Sheep anti-Mouse IgG - Horseradish Peroxidase	GE Healthcare	Cat# NA931; RRID:AB_772210
Goat anti-Rabbit IgG - Peroxidase	Sigma-Aldrich	Cat# A6154; RRID:AB_258284

**Bacterial and virus strains**		

BL21 (DE3) Gold competent ells	Agilent	Cat# 230,132
BL21-CodonPlus (DE3) competent cells	Agilent	Cat# 230,245
DH5α competent cells	Thermo Fisher Scientific	Cat# 18,265,017

**Chemicals, peptides, and recombinant proteins**		

Recombinant His_6_-NSP2	This paper	N/A
Recombinant His_6_-NSP2^1−115^	This paper	N/A
Recombinant His_6_-NSP2^107−212^	This paper	N/A
Recombinant His_6_-NSP2^204−350^	This paper	N/A
Recombinant His_6_-NSP2^G262/265V^	This paper	N/A
Recombinant GIGYF2^743−1,085^	This paper	N/A
Recombinant 4EHP	This paper	N/A
Biotinylated isoxazole	Sigma-Aldrich	Cat# 900,572

**Critical commercial assays**		

Duolink *In Situ* Red Kit	Sigma-Aldrich	Cat# DUO92101
Dual luciferase assay	Promega	Cat# E1960

**Experimental models: Cell lines**		

HEK293T cells	Sigma-Aldrich	Cat# 12,022,001-1VL
Flp-In T-REx HEK293 cells	Thermo Fisher Scientific	Cat# R78007
4EHP^KO^ Flp-In T-REx HEK293 cells	(Jafarnejad et al., 2018)	N/A
GIGYF2^KO^ Flp-In T-REx HEK293 cells	This paper	N/A

**Recombinant DNA**		

Plasmid: pFRT/TO/FLAG/HA-DEST TNRC6C	(Landthaler et al., 2008)	Addgene plasmid #19885
Plasmid: pcDNA5-FRT-TO-FH-NSP2	David Tollervey (Unpublished)	Addgene plasmid #157683
Plasmid: pcDNA5-FRT-TO-FH-NSP2 G262V/G265V	This paper	N/A
Plasmid: pcDNA5-FRT-TO-FH-NSP2^1−212^	This paper	N/A
Plasmid: pcDNA5-FRT-TO-FH-NSP2^1−350^	This paper	N/A
Plasmid: pcDNA5-FRT-TO-FH-NSP2^1−509^	This paper	N/A
Plasmid: pcDNA5-FRT-TO-FH-NSP2^107−638^	This paper	N/A
Plasmid: pcDNA5-FRT-TO-FH-NSP2^204−638^	This paper	N/A
Plasmid: pcDNA5-FRT-TO-FH-NSP2^340−638^	This paper	N/A
Plasmid: pSpCas9(BB)-2A-Puro	(Ran et al., 2013)	Addgene plasmid #62988
Plasmid: pSpCas9(BB)-2A-Puro-*Gigyf2-*sgRNA	This paper	N/A
Plasmid: pET28-His_6_-ZZ	(Barbarin-Bocahu and Graille, 2022)	N/A
Plasmid: pET28-His_6_-ZZ-NSP2	This paper	N/A
Plasmid: pET28-His_6_-ZZ-NSP2 G262V/G265V	This paper	N/A
Plasmid: pET28-His_6_-ZZ-NSP2^1−115^	This paper	N/A
Plasmid: pET28-His_6_-ZZ-NSP2^107−212^	This paper	N/A
Plasmid: pET28-His_6_-ZZ-NSP2^204−350^	This paper	N/A
Plasmid: pCI-Neo	Promega	Cat# E1841
Plasmid: pCI-Neo-V5-GIGYF2	This paper	N/A
Plasmid: pCI-Neo-V5-GIGYF2^1−267^	This paper	N/A
Plasmid: pCI-Neo-V5-GIGYF2^258−495^	This paper	N/A
Plasmid: pCI-Neo-V5-GIGYF2^486−752^	This paper	N/A
Plasmid: pCI-Neo-V5-GIGYF2^743−1,085^	This paper	N/A
Plasmid: pCI-Neo-V5-GIGYF2^1,076-1,320^	This paper	N/A
Plasmid: pCI-Neo-V5-4EHP	This paper	N/A
Plasmid: pCI-Neo-V5-4EHP W95A	This paper	N/A
Plasmid: pGEX-6P-1	Amersham	Cat# 27-4597-01
Plasmid: pGEX-6P-1-4EHP	This paper	N/A
Plasmid: pGEX-6P-1-GIGYF2^743−1,085^	This paper	N/A
Plasmid: pCI-Neo-λNV5-GIGYF2	This paper	N/A
Plasmid: pCI-Neo-λNV5-GW182^SD^	This paper	N/A
Plasmid: pCI-Neo-λNV5-GW182^SD^ ΔPPGL	This paper	N/A
Plasmid: psiCHECK-2	Promega	Cat# C802A
Plasmid: psiCHECK-2-Ifnb1 3′ UTR	(Zhang et al., 2021)	N/A
Plasmid: pCI-Neo-RLuc-5BoxB-A_114_-N_40_-HhR	(Chapat et al., 2017b)	N/A
Plasmid: pCI-Neo-RLuc-5BoxB	(Pillai et al., 2004)	N/A
Plasmid: pCI-Neo-RLuc-6let7a	(Pillai et al., 2004)	N/A
Plasmid: pCI- Neo-FLuc	(Pillai et al., 2004)	N/A

**Software and algorithms**		

Fiji	Fiji	RRID: SCR_002285
Prism 9	GraphPad	RRID:SCR_002798
Illustrator CS6	Adobe	RRID:SCR_010279
ChimeraX	UCSF	RRID:SCR_015872
Image Lab Software	Bio-Rad	RRID:SCR_014210
Jalview	Jalview	RRID:SCR_006459

### Resource availability

#### Lead contact

Further information and requests for resources and reagents should be directed to and will be fulfilled by the lead contact, Clément Chapat (clement.chapat@cnrs.fr).

#### Materials availability

All newly created cell populations generated in this study are available upon request.

### Experimental model and subject details

#### Cell lines and culture

HEK293T cells (Sigma-Aldrich) were routinely maintained in high glucose Dulbecco’s Modified Eagle’s Medium (DMEM) with GlutaMAX supplemented with 10% fetal bovine serum (FBS) and 2% penicillin/streptomycin in a humidified atmosphere of 5% CO2 at 37°C. Flp-In T-REx 293 cells (Thermo Fisher Scientific) were grown in similar conditions supplemented with 100 μg/mL zeocin and 15 μg/mL blasticidin. The absence of mycoplasma contamination in cells was routinely tested. The cell line inducibly expressing Flag-NSP2 was generated by co-transfecting pcDNA5-FRT-TO-FH-Nsp2 (Addgene plasmid 157683) and pOG44 (Thermo Fisher Scientific) with a 1:10 ratio. Transfected cells were selected and maintained in media supplemented with 100 μg/mL hygromycin. Expression of Flag-tagged proteins was induced for 24 h by addition of tetracycline to 1 μg/mL final concentration.

### Method details

#### CRISPR/cas9-mediated genome editing

CRISPR-Cas9-mediated genome editing of HEK293 cells was performed according to Ran et al. The following oligonucleotides encoding a small guide RNA cognate to the coding region of *Gigyf2* gene were used: 5′-CACCGGGAGGAACCCCTTCCACCAT and 5′-AAACATGGTGGAAGGGGTTCCTCCC. These oligos which contain BbsI restriction sites were annealed creating overhangs for cloning of the guide sequence oligos into pSpCas9(BB)-2A-Puro (PX459) V2.0 (Addgene plasmid 62988) by BbsI digestion. To generate KO HEK293 cells, we transfected 700,000 cells with the pSpCas9(BB)-2A-Puro plasmid. 24 h after transfection, puromycin was added in the cell medium to 1.5 μg/mL final concentration. After 72 h, puromycin-resistant cells were isolated into 96-well plates to obtain monoclonal colonies. Clonal cell populations were analyzed by WB for protein depletion.

#### Plasmid cloning

For Flag-NSP2 IP, pcDNA5-FRT-TO-FH-Nsp2 (Addgene plasmid 157683) was used. Truncated versions of NSP2 were generated by replacing the sequence encoding full-length NSP2 by PCR-amplified fragments at the BamHI-XhoI sites of the pcDNA5-FRT-TO-FH vector. The full-length cDNAs encompassing the coding region of human 4EHP and GIGYF2 were obtained by RT-PCR using total RNA from HEK293T cells and cloned into the pCI-Neo vector (Promega) at the XhoI-NotI sites in frame with a sequence encoding a V5 tag inserted at the NheI-XhoI sites. To generate the pCI-λNV5-GIGYF2 vector, a fragment containing the GIGYF2 sequence was obtained by PCR and inserted into the pCI-λNV5 at the XhoI-NotI sites. A similar strategy was used to generate the V5-tagged fragments of GIGYF2 using primers to isolate the following domains encompassing residues: 1–267; 258–495; 486–752; 743-1,085; 1,076-1,320. To construct the pCI-λNV5-GW182^SD^ vector, a fragment encoding the residues 1382–1690 was cloned by PCR as a XhoI-NotI fragment from the pFRT/TO/FLAG/HA-DEST TNRC6C vector (Addgene plasmid 19885) (Landthaler et al., 2008) and inserted into the pCI-λNV5 at the XhoI-NotI sites. For recombinant GST-fused protein expression, full-length 4EHP or GIGYF2^743−1,085^ coding sequences contained in PCR-amplified BamHI-NotI or XhoI-NotI fragments, respectively, were cloned into a pGEX-6P-1 vector (Amersham) in frame with the GST coding sequence. The pET-28b vector (EMD Biosciences) was used to express NSP2 as a recombinant protein fused to an His_6_ tag at the N-terminus. For this, PCR-amplified fragments encoding full-length NSP2 or regions 1–115, 107–212 and 204–350 were inserted at the XhoI-BamHI sites of pET-28b-His_6_-ZZ (Barbarin-Bocahu and Graille, 2022). Amino acid substitutions and deletions were introduced by site-directed mutagenesis using the QuikChange Kit (Agilent). Sequences of the primers used are listed in the Table S1.

#### Extract preparation and immunoprecipitation

Cells were resuspended in a lysis buffer containing 20 mM HEPES-KOH, pH 7.5, 100 mM NaCl, 2.5 mM MgCl2, 0.5% NP40, 0.25% sodium deoxycholate, supplemented with cOmplete EDTA-free protease inhibitor and phosphatase inhibitor Cocktails (Roche), and incubated for 20 min on ice. The lysate was clarified by centrifugation at 10,000 g for 10 min at 4°C. One milligram of extract was used for immunoprecipitation with the indicated antibodies. Thirty microliters of pre-equilibrated Anti-FLAG L5 Magnetic Beads (Thermo Fisher Scientific, A36798) and RNase A (Thermo Fisher Scientific) were added, and the mixtures were rotated overnight at 4°C. Beads were washed five times with lysis buffer and directly resuspended in protein sample buffer for Western blot analysis.

#### Western blot and antibodies

Proteins were separated by SDS-PAGE on 4–15% TGX gradient gels (Bio-Rad) and transferred onto PVDF membranes. The membranes were blocked in PBS containing 5% non-fat milk and 0.1% Tween 20 for 30 min at room temperature. Blots were probed with the following antibodies: rabbit anti-eIF4E2/4EHP (Proteintech, 12227-1-AP), rabbit anti-DDX6 (Proteintech, 14632-1-AP), rabbit anti-CNOT9 (Proteintech, 22503-1-AP), mouse anti-Flag M2 (Sigma-Aldrich, F1804), rabbit anti-GIGYF2 (Proteintech, 24790-1-AP), mouse anti-GAPDH (Proteintech, 60004-1-Ig), mouse anti-V5 tag (Thermo Fisher Scientific, R96025), rabbit anti-ZNF598 (Thermo Fischer Scientific, 703,601) and mouse anti-6xHis Tag (Thermo Fischer Scientific, MA1-21315-HRP).

#### Biotinylated isoxazole (b-isox)-mediated precipitation

HEK293T cells were resuspended in lysis buffer (50 mM HEPES pH 7.5, 150 mM NaCl, 0.1% NP-40, 1 mM EDTA, 2.5 mM EGTA, 10% glycerol, 1 μM DTT) supplemented with cOmplete EDTA-free protease inhibitor, and phosphatase inhibitors, and incubated for 20 min on ice. The lysate was clarified by centrifugation at 10,000 g for 10 min at 4°C. 50 μg of the sample were mixed with 100 μg of b-isox (Sigma-Aldrich) and rotated at 4°C for 90 min. The incubated reaction was then centrifuged at 10,000 g for 10 min to pellet the precipitates. The pellet was washed twice in the lysis buffer and resuspended in protein sample buffer for Western blot analysis. Proteins in the supernatant fractions were precipitated by addition of four volumes of cold acetone, incubated for 1 h at −20°C and centrifuged at 15,000 g for 10 min to pellet the precipitates. The pellet was resuspended in protein sample buffer.

#### Immunofluorescence

WT and GIGYF2^KO^ cells were grown on an 8-well glass slide (Millicell EZ, Merck Millipore Ltd.) and transfected with 100 ng of vector expressing Flag-NSP2. Cells were then fixed in 4% paraformaldehyde for 20 min, washed twice in PBS, permeabilized in PBS containing 0.1% Triton X-100 and 22.5 mg/mL glycine for 5 min. After one wash in PBS, cells were incubated with a blocking solution (PBS containing 0.1% Tween, 22.5 mg/mL glycine, and 1% BSA) for 30 min at room temperature. Primary antibodies diluted in the blocking solution were then added for 1 h at 37°C. After three washes with PBS containing 0.1% Tween (PBS-T), the appropriate secondary antibodies were added for 30 min at 37°C, then washed in PBS-T and mounted on glass slides in a mounting solution containing DAPI (Fluoroshield, Sigma-Aldrich). Fluorescence was visualized under a LEICA-SP8ST-WS confocal microscope. Intensity line scans were performed using Fiji.

#### PLA and confocal microscopy scanning

PLA was performed using the Duolink kit (Sigma-Aldrich) according to manufacturer’s instructions. Transfected cells (100,000) were fixed in 4% paraformaldehyde for 20 min, washed twice in PBS, permeabilized in PBS containing 0.1% Triton X-100 for 5 min and incubated with primary antibodies for 1 h at 37°C in a pre-heated humidity chamber. After two washes in PBS-T, cells were incubated with the appropriate PLA probes for 1 h at 37°C then washed in PBS-T and the ligation solution was added on the coverslips and incubated for 30 min at 37°C. Finally, the amplification solution containing a DNA polymerase was added and incubated with the cells for 100 min at 37°C. For simultaneous immunofluorescence labeling of GIGYF2, secondary antibody coupled with Alexa Fluor 488 (Thermo Fisher Scientific) was added in the amplification solution. After final washes, the cells were mounted on glass slides in a mounting solution with DAPI, and imaging was performed on a LEICA-SP8ST-WS confocal microscope. Quantification of PLA dots per cell was performed using the Fiji software as previously described (Nissan and Parker, 2008). Briefly, the quantification was accomplished by setting a threshold mask with the Otsu Thresholding Filter. Using the “Invert LUT” and “Analyze Particles” tools of Fiji, the PLA dots were automatically counted on the thresholded images. For each cell population, ten pictures with at least 20 cells per picture were used to calculate the mean values. The “Counter cells” plugin was used to analyze the number of cells in each image.

#### Expression and purification of His_6_-NSP2

His_6_-NSP2 was expressed in *E. coli* BL21 (DE3) Gold (Agilent). Large-scale expression was done in 1 L of auto-inducible terrific broth media (ForMedium AIMTB0260) supplemented with kanamycin (50 μg/mL), first at 37°C for 3 h and then at 18°C overnight. The cells were harvested by centrifugation at 4,000 rpm for 30 min and the pellets were resuspended in 30 mL of lysis buffer (50 mM HEPES pH 7.5, 300 mM NaCl, 10 μM ZnCl_2_, 2 mM MgCl_2_ and 20 mM Imidazole) supplemented with one protease inhibitor tablet (Roche), 0.5 mM PMSF and 30 μL benzonase nuclease (Millipore Sigma). The cells were lysed by sonication on ice and the lysate clearance was performed by centrifugation at 20,000 g for 30 min. The supernatant was applied on Ni-NTA resin pre-equilibrated with the lysis buffer, and incubated at 4°C on a rotating wheel for 1 h, followed by a washing step with 30 mL of washing buffer (50 mM HEPES pH 7.5, 1 M NaCl and 10 μM ZnCl_2_). His_6_-NSP2 was eluted by the addition of 15 mL of elution buffer (50 mM Tris/HCl pH 8.0, 300 mM NaCl, 10 μM ZnCl_2_, 2 mM MgCl_2_ and 300 mM Imidazole), followed by concentrating up to 10 mL by a 30 kDa cutoff concentrator. The sample was then diluted to 50 mL using Heparin buffer A (50 mM Tris/HCl pH 8.0, 5 mM β-mercaptoethanol, 10 μM ZnCl_2_), followed by loading on a 5 mL Heparin HP column (Cytiva) and eluted using a NaCl linear gradient from 75 mM (7.5% Heparin buffer B: 50 mM Tris/HCl pH 8.0, 1 M NaCl, 5 mM β-mercaptoethanol, 10 μM ZnCl_2_) to 1 M (100% Heparin buffer B). The fractions containing His_6_-NSP2 protein were collected and concentrated up to 5 mL, followed by sample injection on a Superdex 200 increase 10/300 size-exclusion column (Cytiva) with Gel filtration buffer (50 mM HEPES pH 7.5, 250 mM NaCl, 5 mM β-mercaptoethanol and 10 μM ZnCl_2_). The fractions containing His_6_-NSP2 were collected and concentrated.

#### Expression and purification of 4EHP and GIGYF2^743−1,085^

Expression of GST-4EHP and GST-GIGYF2^743−1,085^ was carried out in *E. coli* BL21 (DE3) Codon+ (Agilent) in 1 L of auto-inducible terrific broth media (ForMedium AIMTB0260) supplemented with ampicillin at 100 μg/mL and chloramphenicol at 25 μg/mL. When the OD_600 nm_ reached 0.6–0.8, cultures were incubated at 20°C for 20 h. Bacteria were harvested by centrifugation and resuspended in lysis buffer (20 mM HEPES pH 7.5, 200 mM NaCl, 5 mM β-mercaptoethanol). Cell lysis was performed by sonication on ice. After centrifugation for 30 min at 20,000 g, 4°C, clarified samples were transferred to batch-bind with Glutathione SepharoseTM 4B (Cytiva) resin for ∼1 h at 4°C followed by a washing step with 30 mL of washing buffer (20 mM HEPES pH 7.5, 1 M NaCl and 5 mM β-mercaptoethanol) supplemented with 10 mM ATP. 3C protease digestion was performed overnight at 4°C to remove GST. The untagged GIGYF2^743−1,085^ domain was present in the flowthrough and further purified on an HiTrap S FF column (Cytiva) using a linear gradient of 92.5% Hitrap buffer A (20 mM HEPES pH7.5, 5 mM β-mercaptoethanol) to 100% Hitrap buffer B (20 mM HEPES pH 7.5, 1 M NaCl and 5 mM β-mercaptoethanol). The peak fractions corresponding to 4EHP and GIGYF2^743−1,085^ were pooled, concentrated and used for pull-down assays.

#### Ni–NTA pull-down assays

Pull-down experiments were performed by incubating 1 nmol of His_6_-NSP2, FL or truncated, with equimolar amount of untagged GIGYF2^743−1,085^ or 4EHP, or GST used as control. All proteins were free of nucleic acids according to the OD_280 nm_/OD_260 nm_ ratio. Binding buffer (25 mM HEPES pH 7.5, 200 mM NaCl, 50 mM imidazole, 10% glycerol and 0.1% Triton X-100) was added to a final volume of 60 μL. The reaction mixtures were incubated on ice for 1 h. After that, 10 μL was withdrawn and used as an input fraction for SDS–PAGE analysis. The remaining 50 μL were incubated at 4°C for 2 h with 40 μg of HisPur Ni–NTA magnetic beads (Thermo Scientific) pre-equilibrated in binding buffer, in a final volume of 200 μL. After binding, beads were washed three times with 500 μL of binding buffer. Bound proteins were eluted with 50 μL of elution buffer (20 mM HEPES pH 7.5, 200 mM NaCl, 250 mM imidazole, 10% glycerol and 0.1% Triton X-100). Samples were resolved on SDS–PAGE and visualized by Coomassie blue staining.

#### Co-expression and GST pull-down between His_6_-NSP2 and GST-GIGYF2^743−1,085^

Full-length His_6_-NSP2 and GST-GIGYF2^743−1,085^ proteins were co-expressed in BL21 (DE3) Gold *E. coli* (Agilent technologies). Small-scale expression was done in 5 mL of auto-inducible terrific broth media (ForMedium, AIMTB0260) supplemented with ampicillin (100 μg/mL) and kanamycin (50 μg/mL), first at 37°C for 3 h and then at 18°C overnight. The cells were harvested by centrifugation at 4,000 rpm for 30 min and the pellets were resuspended in 750 μL of lysis buffer (50 mM HEPES pH 7.5, 200mM NaCl, 5 mM β-mercaptoethanol). The cells were lysed by sonication on ice and the lysate clearance was performed by centrifugation at 13,000 g for 30 min. The supernatant was applied on Glutathione SepharoseTM 4B resin (Cytiva) pre-equilibrated with the lysis buffer, incubated at 4°C on a rotating wheel for 1 h, followed by a washing step with 1 mL of lysis buffer (50 mM HEPES pH 7.5, 200mM NaCl, 5 mM β-mercaptoethanol). Retained proteins were eluted by 50 μL of elution buffer (50 mM HEPES pH 7.5, 200mM NaCl, 5 mM β-mercaptoethanol, 20mM GSH), followed by SDS-PAGE and Coomassie blue staining or Western blot.

#### Tethering and luciferase assays

Tethering assays were performed as previously described (Chapat et al., 2017a, 2017b). Briefly, HEK293T cells were transfected with 20 ng of RLuc-5BoxB (or RLuc-5BoxB-A_114_-N_40_-HhR), 5 ng of FLuc, and 100 ng of λN-fusion constructs per well in a 24-well plate by using Lipofectamine 2000 (Thermo Scientific) according to the manufacturer’s instructions. 100 ng of vectors encoding Flag-NSP2 or Flag (empty vector) were also added in the transfection mixture. Cells were lysed 24 h after transfection and luciferase activities were measured with the Dual-Luciferase Reporter Assay System (Promega) in a GloMax 20/20 luminometer (Promega). RL activity was normalized to the activity of co-expressed FL, and the normalized RL values are shown as repression fold relative to the indicated control. For experiments with miRNA reporters, HEK293T were co-transfected in a 24-well plate with 100 ng of vectors encoding Flag-NSP2, or Flag (empty vector) as control, 20 ng of RLuc-*6let7a* and 5 ng of FLuc plasmid. For the reporter containing the 3′UTR of *Ifnb1*, 20 ng of psiCHECK2-RLuc-*Ifnb1* 3′ UTR reporter (Zhang et al., 2021), or empty psiCHECK2 (Promega), was added along with 100 ng of vectors encoding Flag-NSP2 or Flag (empty vector).

### Quantification and statistical analysis

Statistical analyses were performed with GraphPad Prism software for Windows. All data were calculated by two-tailed Student’s t-test and presented as mean ± standard deviation (SD). p values less than 0.001, 0.01 and 0.05 were assigned with ∗∗∗, ∗∗ and ∗, respectively.

## Data Availability

• All data reported in this paper will be shared by the lead contact upon request. • This paper does not report original code. • Any additional information required to reanalyze the data reported in this paper is available from the lead contact upon request.
