# Supplementary material for: The SARS-CoV-2 protein NSP2 impairs the silencing capacity of the human 4EHP-GIGYF2 complex
Source: iScience. 2022 Jun 20;25(7):104646. doi: 10.1016/j.isci.2022.104646 (PMC9213009; doi:10.1016/j.isci.2022.104646)
Supplement: Document S1. Figures S1–S4 and Table S1 [file mmc1.pdf]

iScience, Volume *25*

Supplemental information

The SARS-CoV-2 protein NSP2 impairs the silencing capacity of the human 4EHP-GIGYF2 complex

Limei Zou, Clara Moch, Marc Graille, and Clément Chapat

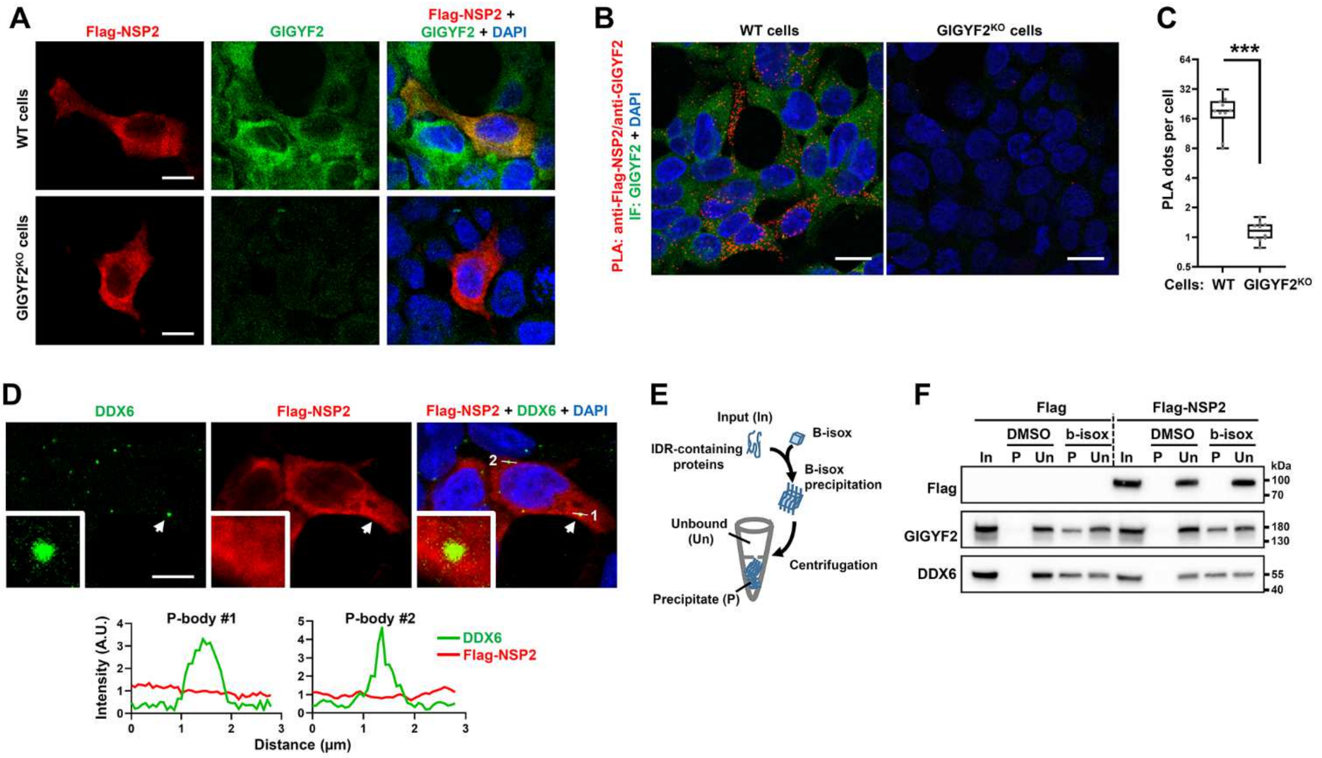

Figure S1. NSP2 associates with the cytoplasmic form of GIGYF2, related to Figure 1. (**A**) Immunofluorescence analysis of WT and GIGYF2^{KO} HEK293 cells expressing Flag-NSP2 and immunostained for the Flag tag (red) and GIGYF2 (green). Nuclei were stained with DAPI (blue). Images were acquired under identical conditions at objective X63. Z-projection of 3 stacks (0.35 µm each). Scale bar, 10 µm. (**B**) Proximity Ligation Assay between Flag-NSP2 and endogenous GIGYF2. WT and GIGYF2^{KO} HEK293 cells were transfected with vector expressing Flag-NSP2, and PLA was performed using anti-Flag and anti-GIGYF2 antibodies. Representative images of PLA (red), along with GIGYF2 immunofluorescence (IF; green) and DAPI (blue) are shown. Z-projection of 3 stacks (0.35 µm each). Scale bar, 15 µm. (**C**) Effect of GIGYF2 KO on PLA signals. The number of PLA dots per cell (n>270) was quantified (mean±S.D.) in Flag-NSP2-expressing WT and GIGYF2^{KO} HEK293 cells using Fiji software. For each cell population, ten pictures with at least 20 cells per picture were used to calculate the mean values (±SD), and the P value was determined by two-tailed Student's t-test. (***) $P < 0.001$. (**D**) Confocal analysis of HEK293T cells expressing Flag-NSP2 and immunostained for the Flag tag (red) and DDX6 (green). Nuclei were stained with DAPI (blue). Insets show a higher magnification of a P-body (arrow). White lines in composite images show line scans used to determine the distribution of both DDX6 and Flag-NSP2 signals. Line plot graphs (bottom) show integrated intensity values for the two indicated DDX6 foci. Z-projection of 3 stacks (0.35 µm each). A.U., Arbitrary Unit. Scale bar, 10 µm. (**E**) Schematic overview of the biotinylated isoxazole (b-isox)-mediated precipitation of Intrinsically-Disordered Region (IDR)-containing proteins. (**F**) B-isox precipitation of endogenous GIGYF2 in Flag-NSP2 expressing HEK293T cell extract. Cells transfected by an empty vector (Flag) were used as a negative control. Input (In), Precipitated (P) and Unbound (Un) fractions were analyzed by Western blot with the indicated antibodies. DMSO, used as the solubilizing agent for b-isox, served as a mock control.

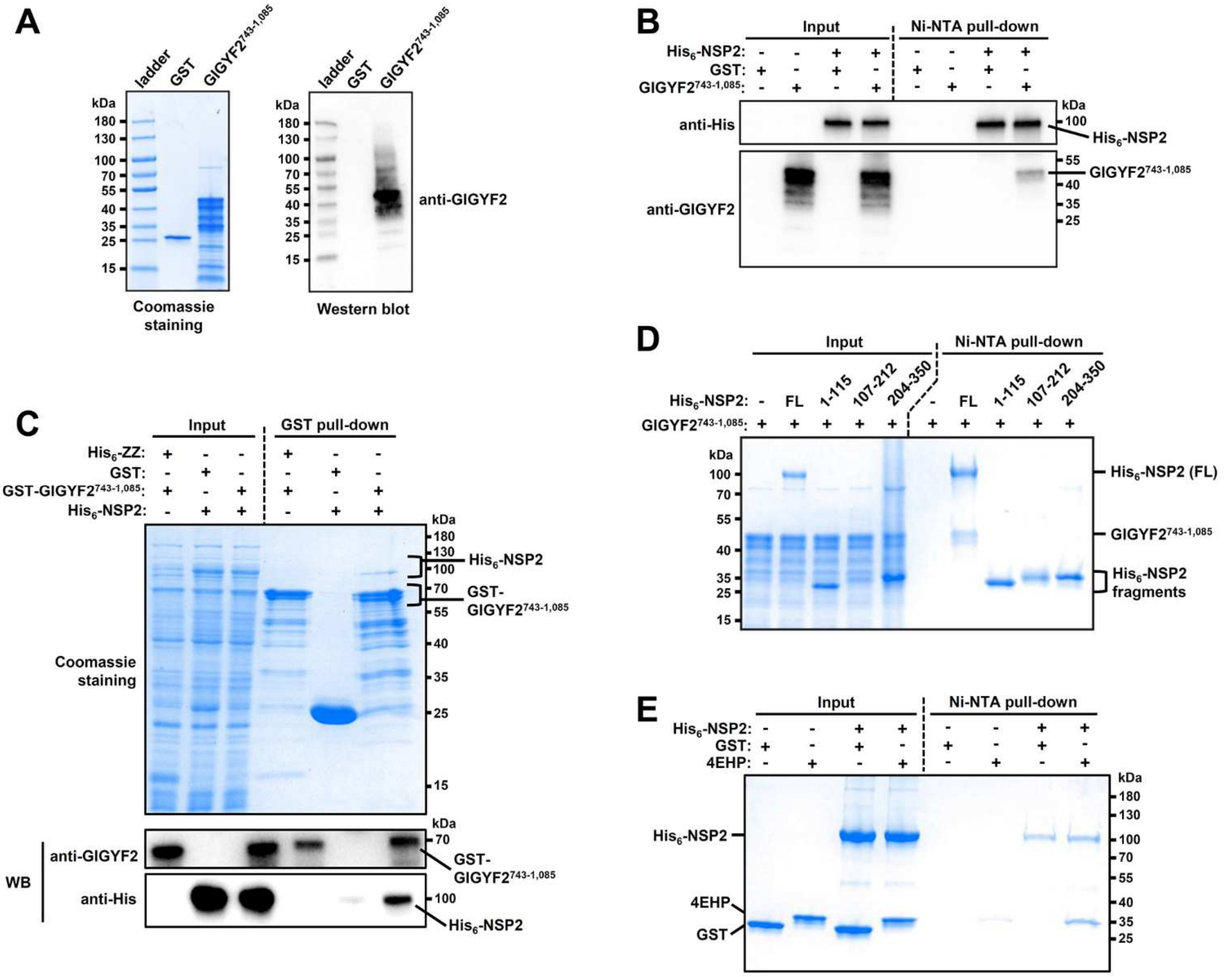

Figure S2. His$_6$-NSP2 binds GIGYF2$^{743\text{-}1,085}$ and 4EHP *in vitro*, related to Figure 2. (**A**) Recombinant GIGYF2$^{743\text{-}1,085}$ is recognized by an antibody raised against GIGYF2. Recombinant GST and GIGYF2$^{743\text{-}1,085}$ proteins were analyzed by SDS-PAGE followed by Coomassie blue staining (left panel), or by Western blot with an antibody raised against the 756-1,104 region of GIGYF2 (right panel). GST served as negative control. (**B**) Ni-NTA pull-down assay showing the interaction between recombinant His$_6$-NSP2 and untagged GIGYF2$^{743\text{-}1,085}$ by Western blot. The starting material (Input) and bound (Ni-NTA pull-down) fractions used in Figure 2c were diluted (1/30) and analyzed by Western blot with the indicated antibody. (**C**) GST pull-down assay showing the interactions between recombinant GST-fused GIGYF2$^{743\text{-}1,085}$ and His$_6$-NSP2. Both His$_6$-NSP2 and GST-GIGYF2$^{743\text{-}1,085}$ were co-expressed in *E.coli* and GST pull-down assays were performed using an *E. coli* lysate. The starting material (Input) and bound fractions were analyzed by SDS-PAGE followed by Coomassie blue staining and Western blot with the indicated antibodies. GST served as negative control. (**D**) Recombinant GIGYF2$^{743\text{-}1,085}$ interacts with full-length His$_6$-NSP2, but not with truncated versions. The following regions of His$_6$-NSP2 were used: residues 1-115, 107-212 and 204-350. Ni-NTA pull-down assays were performed with the indicated recombinant proteins. The starting material (Input) and bound (Ni-NTA pull-down) fractions were analyzed by SDS-PAGE followed by Coomassie blue staining. (**E**) Ni-NTA pull-down assay showing the interaction between recombinant His$_6$-NSP2 and untagged full-length 4EHP. GST served as negative control and the indicated extracts were analyzed by SDS-PAGE followed by Coomassie blue staining.

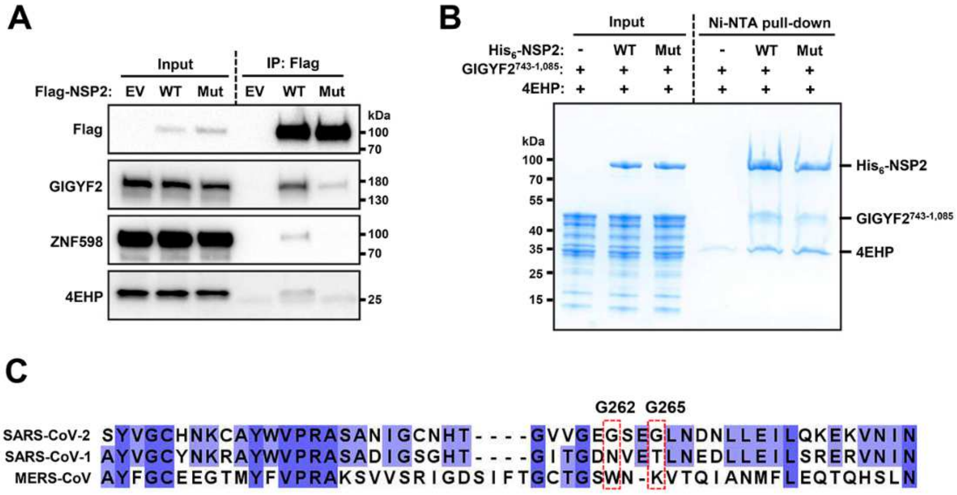

Figure S3. The G262V/G265V natural variation of NSP2 reduces its interaction with 4EHP-GIGYF2 *in cellulo*, not *in vitro*, related to Figure 2. (**A**) The G262V/G265V natural variation of NSP2 impairs its interaction with endogenous 4EHP-GIGYF2 by co-IP. WT and G262V/G265V (Mut) Flag-NSP2 were transitory expressed in HEK293T, and Flag IP were performed with RNase A-treated extracts. The starting material (Input) and bound fractions were analyzed by Western blot. EV: empty vector. (**B**) Ni-NTA pull-down assay showing the interaction between the WT or G262V/G265V version (Mut) of His_6-NSP2 with both untagged 4EHP and $GIGYF2^{743\text{-}1,085}$. The starting material (Input) and bound (Ni-NTA pull-down) fractions were analyzed by SDS-PAGE followed by Coomassie blue staining. (**C**) Alignment of SARS-CoV-2 NSP2 (region: 232-281) with its SARS-CoV-1 (GenBank QJE50587.1) and MERS-CoV (GenBank QFQ59585.1) homologous versions. The sequences were aligned with Clustal Omega using default parameters, and colored in Jalview by identity.

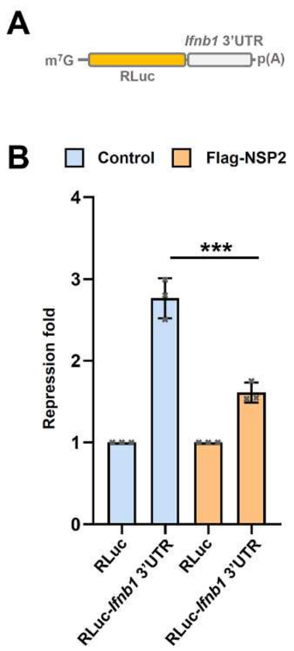

Figure S4. NSP2 derepresses the expression of a reporter harboring the *Ifnb1* 3′ UTR, related to Figure 4. **(A)** Schematic of the psiCHECK2-RLuc-*Ifnb1* 3′ UTR reporter. **(B)** HEK293T cells were co-transfected with psiCHECK2-RLuc-*Ifnb1* 3′ UTR reporter or the psiCHECK2 reporter (as control). Vectors encoding Flag-NSP2 or Flag (empty vector) were also added in the transfection mixture. Luciferase activity was measured 24 h after transfection. RLuc values were normalized against FLuc levels, and the repression fold was calculated for the psiCHECK2-RLuc-*Ifnb1* 3′ UTR reporter relative to the psiCHECK2 reporter level for each condition. Data are presented as mean ± SD (n = 3). (***) $P < 0.001$ (two-tailed Student's t test).

Table S1. Oligonucleotides used in this study, related to STAR Methods.

Fragments		Sequences* (5'→3')
XhoI-GIGYF2-NotI	Forward	GAGCTCGAGATGGCAGCGGAAACGCAGAC
	Reverse	GAGGCGGCCGCTCAGTAGTCATCCAACGTCTC
XhoI-GIGYF2$^{1-267}$-NotI	Forward	GAGCTCGAGATGGCAGCGGAAACGCAGAC
	Reverse	GAGGCGGCCGCCTACCAGCCTGCAGAACGAGGGC
XhoI-GIGYF2$^{258-495}$-NotI	Forward	GAGCTCGAGAGTCCTGATGGCCCTCGTTC
	Reverse	GAGGCGGCCGCCTAACCAGGAGCACCTACAACTG
XhoI-GIGYF2$^{486-752}$-NotI	Forward	GAGCTCGAGGTTGAAACACCAGTTGTAGG
	Reverse	GAGGCGGCCGCCTACTTCTCTAGCTGCTGAAGCTG
XhoI-GIGYF2$^{743-1,085}$-NotI	Forward	GAGCTCGAGGCCCTGGAACAGCTTCAGCAG
	Reverse	GAGGCGGCCGCCTAACTACTGACTAGGTCAGATGC
XhoI-GIGYF2$^{1,076-1,320}$-NotI	Forward	GAGCTCGAGAACCAGTGGGCATCTGACCT
	Reverse	GAGGCGGCCGCTCAGTAGTCATCCAACGTCTC
BamHI-NSP2-XhoI	Forward	AAAGGATCCATGGCATACACACGCTATGTTG
	Reverse	AACTCGAGCTAGGCACCTCCCTTGAGCGTAA
BamHI-NSP2$^{1-115}$-XhoI	Forward	AAAGGATCCATGGCATACACACGCTATGTTG
	Reverse	AACTCGAGCTAACCGTCCAGCTTCTTTTTCTCCAC
BamHI-NSP2$^{107-212}$-XhoI	Forward	AAAGGATCCAGAGTGGAGAAAAAGAAGCTGGAC
	Reverse	AACTCGAGCTAACCAGATTCGTTGTGATATTCAGC
BamHI-NSP2$^{204-350}$-XhoI	Forward	AAAGGATCCCTCGCTGAATATCACAACGAATCT
	Reverse	AACTCGAGCTAGAGTATGCTTTTCTGTTCACCAAT
BamHI-NSP2$^{1-212}$-XhoI	Forward	AAAGGATCCATGGCATACACACGCTATGTTG
	Reverse	AACTCGAGCTAACCAGATTCGTTGTGATATTCAGC
BamHI-NSP2$^{1-350}$-XhoI	Forward	AAAGGATCCATGGCATACACACGCTATGTTG
	Reverse	AACTCGAGCTAGAGTATGCTTTTCTGTTCACCAAT
BamHI-NSP2$^{1-509}$-XhoI	Forward	AAAGGATCCATGGCATACACACGCTATGTTG
	Reverse	AACTCGAGCTAACACAGAGCCAAGAACTTATTC
BamHI-NSP2$^{107-638}$-XhoI	Forward	AAAGGATCCAGAGTGGAGAAAAAGAAGCTGGAC
	Reverse	AACTCGAGCTAGGCACCTCCCTTGAGCGTAA
BamHI-NSP2$^{204-638}$-XhoI	Forward	AAAGGATCCCTCGCTGAATATCACAACGAATCT
	Reverse	AACTCGAGCTAGGCACCTCCCTTGAGCGTAA
BamHI-NSP2$^{340-638}$-XhoI	Forward	AAAGGATCCGCTTGGAATATTGGTGAACAGAAA
	Reverse	AACTCGAGCTAGGCACCTCCCTTGAGCGTAA
NSP2 G262V/G265V mutant	Forward	GTGGTCGGGGAAGTGAGCGAGGTTCTGAATGATAACC
	Reverse	GGTTATCATTCAGAACCTCGCTCACTTCCCCGACCAC
BamHI-4EHP-NotI	Forward	GAGGGATCCAACAACAAGTTCGACGCTTTG
	Reverse	GAGGCGGCCGCTCATGGCACATTCAACCGCG
XhoI-4EHP-NotI	Forward	AACTCGAGAACAACAAGTTCGACGCTTTG
	Reverse	AAGCGGCCGCTCATGGCACATTCAACCGC
4EHP W95A mutant	Forward	GCCTCTGTGGAGCAGTTCGCGAGGTTTTATAGCCACATG
	Reverse	CATGTGGCTATAAAACCTCGCGAACTGCTCCACAGAGGC
XhoI-GW182^{SD}-NotI	Forward	AACTCGAGAGCATCAACTGGCCCCCAG
	Reverse	AAGCGGCCGCTTACAGGGACTCCCCGCTGA
GW182^{SD} ΔPPGL mutant	Forward	CTGCACCCACGAGGCCAACCAATCCCAAG
	Reverse	CTTGGGATTGGTTGGCCTCGTGGGTGCAG

*Restriction sites in the oligonucleotides are underlined.
